# Prognostic significance of pre-treatment ALBI grade in advanced non-small cell lung cancer receiving immune checkpoint therapy

**DOI:** 10.1038/s41598-021-94336-9

**Published:** 2021-07-23

**Authors:** Ryosuke Matsukane, Hiroyuki Watanabe, Kojiro Hata, Kimitaka Suetsugu, Toshikazu Tsuji, Nobuaki Egashira, Yoichi Nakanishi, Isamu Okamoto, Ichiro Ieiri

**Affiliations:** 1 Department of Pharmacy, Kyushu University Hospital, 3-1-1 Maidashi, Higashi-ku, Fukuoka, 812-8582 Japan; 2 Department of Clinical Pharmacology and Biopharmaceutics, Graduate School of Pharmaceutical Sciences, Kyushu University, Fukuoka, Japan; 3 Research Institute for Diseases of the Chest, Graduate School of Medical Sciences, Kyushu University, Fukuoka, Japan

**Keywords:** Prognostic markers, Non-small-cell lung cancer, Cancer immunotherapy

## Abstract

The liver is an essential organ for regulating innate and acquired immunity. We hypothesized that the pre-treatment hepatic function affects the clinical outcome of immune checkpoint inhibitors (ICIs) in non-small cell lung cancer (NSCLC). We analyzed 140 patients with NSCLC who received ICIs. We investigated the association between pre-treatment liver function, assessed using the albumin–bilirubin (ALBI) grade, and clinical outcomes in univariate, multivariate, and propensity score matching analyses. Patients were divided into four grades according to pre-treatment liver function. Eighty-eight patients had good hepatic reserve (ALBI grade 1 or 2a), whereas 52 patients had poor hepatic reserve (ALBI grade 2b or 3). In the univariate Kaplan–Meier analysis, the ALBI grade 1, 2a group had a significantly prolonged progression-free survival (PFS, 5.3 versus 2.5 months, *p* = 0.0019) and overall survival (OS, 19.6 vs. 6.2 months, *p* = 0.0002). These results were consistent, regardless of whether the analysis was performed in patients with a performance status of 0 or 1 at pre-treatment (N = 124) or in those selected using propensity score matching (N = 76). In the multivariate analysis, pre-treatment ALBI grade was an independent prognostic factor for both PFS (hazard ratio [HR] 0.57, 95% confidence interval [95% CI] 0.38–0.86, *p* = 0.007) and OS (HR 0.45, 95% CI 0.29–0.72, *p* = 0.001). Our results suggest that pre-treatment hepatic function assessed by ALBI grade could be an essential biomarker for predicting the efficacy of treatment with ICIs in NSCLC.

## Introduction

Immune checkpoint inhibitors (ICIs) exert antitumor effects by inhibiting programmed cell death-1 (PD-1) and programmed cell death ligand (PD-L1), which regulate tumor immune tolerance^1,2^. With the advent of anti-PD-1 and anti-PD-L1 antibodies, ICIs have become the standard therapy for non-small cell lung cancer (NSCLC) and have improved prognosis^3,4^. Moreover, as the safety and efficacy of ICI are established, its use in patients with comorbidities has been expanded in clinical practice. Therefore, the effects of common underlying diseases, such as hypertension, type 2 diabetes, liver impairment, and renal dysfunction, which might affect the immune function or drug pharmacokinetics physiologically, should be considered; however, only a few studies have investigated the effects of comorbidities on ICI therapy.

The liver is an essential organ for protein production and regulation of innate and acquired immunity^5^. Previous research showed that liver impairment affects the interaction among various cytokines, which are necessary for immune activation, and causes the fluctuation of T cell subset repertoires^6,7^. In a recent real-world analysis of the treatment of NSCLC with nivolumab, liver metastasis was described as an independent poor prognostic factor. However, the liver is not a frequent site of metastasis in NSCLC, unlike the brain and bone^8^. Thus, the hepatic reserve or liver dysfunction might need to be considered for treatment with ICIs in NSCLC.

Liver function is currently graded using the Child–Pugh score, which was initially developed for assessing prognosis in patients with cirrhosis. The score considers five clinical measures: serum albumin, total bilirubin levels, prothrombin time, extent of ascites, and the evaluation of hepatic encephalopathy. However, several problems have been noted; for instance, the assessment of ascites and encephalopathy is subjective, and serum albumin levels correlate with the extent of ascites^9^. In addition, when we assess patients with a mild liver dysfunction and not cirrhosis, most of them are classified as grade A. Therefore, an alternative grading system called the albumin–bilirubin (ALBI) score has been reported in prior studies on hepatocellular carcinoma (HCC)^9–12^. The ALBI score is an objective, statistically calculated score based on serum albumin and total bilirubin levels, and it is a predictor of life expectancy in HCC^9^.

In this study, we retrospectively investigated the effect of hepatic impairment, assessed with the ALBI score, on the prognosis and safety of ICI treatment in NSCLC.

## Materials and methods

### Study design

We retrospectively analyzed patients with unresectable, metastatic, and recurrent NSCLC treated with ICIs at Kyushu University Hospital. Patients treated with at least one infusion of nivolumab, pembrolizumab, and atezolizumab between January 2016 and October 2019 were eligible. Patients who received ICIs in a part of other clinical trials were excluded from this study. The patients were followed up throughout the clinical course for at least nine months, and the cutoff date for data collection was July 31, 2020. We extracted clinical information, social history, and clinical laboratory data at pre-treatment from electronic medical records. Pre-treatment laboratory data were obtained just before the first administration of ICI or at least within three weeks. Based on the questionnaire given to the patients, we decided on the smoking and drinking habitant. The drinking history was classified into four categories: daily drinking, drinking 2–3 times a week, occasional drinking, and no drinking habit. Patients who drank more than 2–3 times a week were classified as heavy to moderate, and those who drank occasionally or had no drinking habits were classified as occasional/never. Grading of immune-related adverse events (irAEs) was conducted using the National Cancer Institute Common Terminology Criteria for Adverse Events v.5.0.

### Assessment of ALBI score and grade

The ALBI score was calculated using the serum albumin and bilirubin levels, with the following equation: ALBI score = (log10 bilirubin [µmol/L] × 0.66) + (albumin [g/L] ×  − 0.085)^9^. The ALBI score was divided into four grades according to a previous study: grade 1 (ALBI score ≤  − 2.60), grade 2a (− 2.60 < ALBI score <  − 2.27), grade 2b (− 2.27 ≤ ALBI score ≤  − 1.39), and grade 3 (− 1.39 < ALBI score)^9,12^.

### Assessment of nutrition and immune parameters

The modified Glasgow prognostic score (mGPS) was calculated using the categorical classification of serum C-reactive protein (CRP) and serum albumin levels^13^. Patients with CRP > 5 mg/L and albumin < 35 g/L were given an mGPS of 2. Patients with CRP > 5 mg/L or albumin < 35 g/L were given an mGPS of 1. Patients who did not satisfy either criterion received an mGPS of 0. The prognostic nutritional index (PNI) was calculated using the following equation: serum albumin [g/L] + 0.005 × total lymphocyte counts in peripheral blood (per µL)^14^. The neutrophil-to-lymphocyte ratio (NLR) was calculated by dividing the number of neutrophils by the number of lymphocytes^15^.

### Statistical analysis

The association between ALBI grade and patient clinical variables was examined with Fisher’s exact test. The association between ALBI grade and laboratory data was examined with the Mann–Whitney U test. A receiver operating characteristic (ROC) curve analysis was used to assess the ALBI score and other factors. Cutoff values were determined using the highest Youden index. For survival analysis, follow-up time was defined from the first dose to the date of last known contact or death. Survival probabilities and median survival, with 95% confidence intervals (CI), were analyzed using the Kaplan–Meier method, and different groups were compared using the log-rank test. A multivariate analysis of survival outcome and patient characteristics was performed using the Cox proportional hazards model. The results were expressed as hazard ratio (HR) with 95% CI, and p values were calculated with the Wald test. The propensity score was calculated using the logistic regression model that comprised the following baseline characteristics as covariates: age, sex, liver metastasis, drinking history, number of prior treatment regimens, and performance status (PS). Propensity score matching was performed as one-to-one matching between the ALBI grade 1, 2a group and the ALBI 2b, 3 groups with the nearest neighbor matching, using a 0.2 caliper width. Throughout the study, statistical tests were two-sided, and p values less than 0.05 were considered significant. All quantification, calibration, and statistical analyses were carried out using GraphPad Prism version 9.0.0 (GraphPad Software, La Jolla, CA, USA) and JMP version 15.1.0 (SAS Institute, Inc., Cary, NC, USA).

### Ethics approval

This study was conducted in accordance with the Declaration of Helsinki. The requirement for informed consent was waived owing to the study’s retrospective nature, and our official website was used as an opt-out method. Ethical approval was provided by the Institutional Review Boards of the Kyushu University Graduate School and Faculty of Medicine (Approval No. 2020-155).

## Results

### Background characteristics of patients

We collected the data of 140 patients with advanced or recurrent NSCLC who were treated with ICIs, as summarized in Table 1. The median age of the patients was 66 (range, 36–88) years, and 107 (76.4%) patients were male. A total of 124 (88.6%) patients had a PS of 0–1, 93 (66.4%) had adenocarcinomas, and 65 (46.5%) had a PD-L1 expression above 1%. Among all patients, the hepatic reserve at pre-treatment was classified with the modified ALBI grade as follows: 52 (37.1%) had grade 1, 36 (25.7%) had grade 2a, 45 (32.2%) had grade 2b, and 7 (5.0%) had grade 3. As a feature of hepatic impairment, gammaglobulinemia was evaluated using the albumin-globulin ratio, which correlated with the ALBI score change (Supplementary Fig. 1).

**Table 1:** Patients’ background characteristics (n = 140).

Characteristic	No. of patients (%)	
**Age, median—years (range)**	66	(36–88)
**Sex—no. (%)**		
Male	107	(76.4)
Female	33	(23.6)
**Smoking history—no. (%)**		
Current or former	111	(79.3)
Never	29	(20.7)
**Drinking history—no. (%)**		
Heavy/moderate	62	(44.3)
Occasional/never	78	(55.7)
**ECOG PS—no. (%)**		
0–1	124	(88.6)
≥ 2	16	(11.4)
**Common sites of metastasis—no. (%)**		
Brain	46	(32.9)
Bone	46	(32.9)
Lung	29	(20.7)
Liver	13	(9.3)
**Histology—no. (%)**		
Adenocarcinoma	93	(66.4)
Squamous	36	(25.7)
Other	11	(7.9)
**PD-L1 status—no. (%)**		
TPS ≥ 50%	42	(30.0)
TPS 1–49%	23	(16.5)
TPS < 1%	31	(22.1)
not investigated	44	(31.4)
**ICI line of treatment—no. (%)**		
First line	36	(25.7)
Second line	46	(32.9)
Third line or more	58	(41.4)
**Administered ICIs—no, (%)**		
Nivolumab	71	(50.7)
Pembrolizumab	43	(30.7)
Pembrolizumab + chemotherapy	9	(6.4)
Atezolizumab	17	(12.2)
**Modified ALBI grade—no, (%)**		
Grade 1	52	(37.1)
Grade 2a	36	(25.7)
Grade 2b	45	(32.2)
Grade 3	7	(5.0)

*ECOG PS* eastern cooperative oncology group performance status, *PD-L1* programmed cell death ligand-1, *ICI* immune-checkpoint inhibitor, *ALBI grade* albumin–bilirubin grade, *TPS* tumor proportion score.

### Association between ALBI grade and survival in univariate analysis

**Figure 1:**
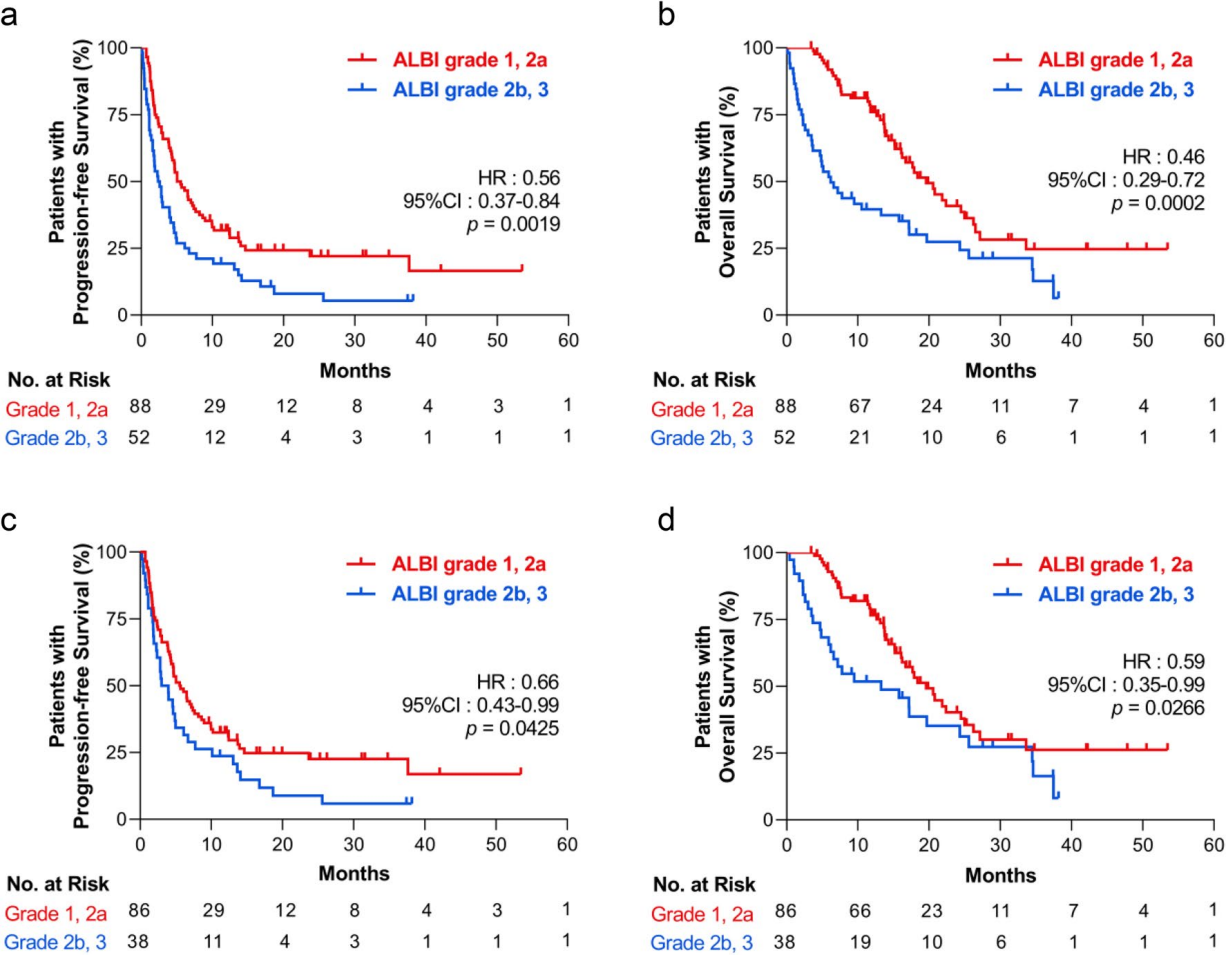
Kaplan–Meier survival analysis of patients with non-small cell lung cancer receiving immune checkpoint inhibitors, according to their pre-treatment ALBI grade. (**a**) Progression-free survival (PFS) and (**b**) overall survival (OS) of all patients (N = 140). (**c**) PFS and (**d**) OS curve in patients with a performance status of 0 and 1 at pre-treatment (N = 124). ALBI, albumin–bilirubin; HR, hazard ratio; CI, confidence interval.

We initially performed ROC analysis for the pre-treatment ALBI score and 6-month progression-free survival (PFS) and 6-month overall survival (OS) to evaluate the feasibility and cutoff value. The analysis showed that the ALBI score significantly estimated both PFS (area under the ROC curve [AUC] 0.60, sensitivity 85.7%, specificity 36.9%, *p* = 0.0489) and OS (AUC 0.74, sensitivity 77.9%, specificity 69.4%, *p* < 0.0001). The superior cutoff value for OS was − 2.22, which was in close conformity with the modified ALBI grade 2a and 2b boundaries (− 2.27). Thus, we classified the patients as follows: pre-treatment ALBI grade 1 and 2a for a better hepatic reserve and ALBI grade 2b and 3 for the worse group. Results of the analysis of the association between the hepatic reserve and patient characteristics are shown in Table 2. In the univariate Kaplan–Meier analysis, the median PFS in the ALBI grade 1, 2a group was 5.3 months and that in the grade 2b, 3 group was 2.5 months (HR 0.56, 95% CI 0.37–0.84, *p* = 0.0019, Fig. 1a). The median OS rates were 19.6 and 6.6 months, respectively (HR 0.46, 95% CI 0.29–0.72, *p* = 0.0002, Fig. 1b). Thus, patients with a superior ALBI grade showed significantly prolonged survival. In both groups, a lower serum albumin level was observed in the ALBI grade 2b, 3 group, but there was no difference in total bilirubin levels (Supplementary Fig. 2a). White blood cell and platelet counts, and CRP levels were increased, and red blood cell count and hemoglobin levels decreased in the ALBI grade 2b, 3 group (Supplementary Fig. 2b). Although alkaline phosphatase significantly increased in the ALBI grade 2b, 3 group, other liver-related parameters showed no differences (Supplementary Fig. 2c).

**Table 2:** Association between ALBI Grade and clinical variables.

Characteristic	ALBI Grade 1, 2a		ALBI Grade 2b, 3		*p*
	(n = 88)		(n = 52)		
**Age, median—years (range)**	66	(36–88)	67	(41–84)	
< 70	55	(62.5)	35	(67.3)	0.590
≥ 70	33	(37.5)	17	(32.7)	
**Sex—no. (%)**					
Male	65	(73.9)	42	(80.8)	0.414
Female	23	(26.1)	10	(19.2)	
**Smoking history—no. (%)**					
Never	20	(22.7)	9	(17.3)	0.521
Current or former	68	(77.3)	43	(82.7)	
**Drinking history—no. (%)**					
Occasional/never	50	(56.8)	28	(53.8)	0.860
Heavy/moderate	38	(43.2)	24	(46.2)	
**ECOG PS—no. (%)**					
0–1	86	(97.7)	38	(73.1)	< 0.0001
≥ 2	2	(2.3)	14	(26.9)	
**Common sites of metastasis—no. (%)**					
Brain	30	(34.1)	16	(30.8)	0.714
Bone	22	(25.0)	24	(46.2)	0.015
Lung	18	(20.5)	11	(21.2)	> 0.999
Liver	8	(9.1)	5	(9.6)	> 0.999
**Histology—no. (%)**					
Adenocarcinoma	60	(68.2)	33	(63.5)	0.583^a^
Squamous	22	(25.0)	14	(26.9)	
Other	6	(6.8)	5	(9.6)	
**PD-L1 status—no. (%)**					
TPS ≥ 50%	22	(25.0)	20	(38.4)	0.381^b^
TPS 1–49%	16	(18.2)	7	(13.5)	
TPS < 1%	24	(27.3)	7	(13.5)	
Not investigated	26	(29.5)	18	(34.6)	
**ICI line of treatment—no. (%)**					
First and second line	46	(52.3)	36	(69.2)	0.053
Third line or more	42	(47.7)	16	(30.8)	
**Administered ICIs—no, (%)**					
Nivolumab	47	(53.4)	24	(46.2)	0.485^c^
Pembrolizumab	21	(23.9)	22	(42.3)	
Pembrolizumab + chemotherapy	7	(7.9)	2	(3.8)	
Atezolizumab	13	(14.8)	4	(7.7)	
**Baseline steroid use—no, (%)**					
Yes	5	(5.7)	5	(9.6)	0.500
No	83	(94.3)	47	(90.4)	
**Laboratory tests—median, (IQR)**					
Albumin	3.9	(3.7–4.1)	3.0	(2.6–3.3)	< 0.001
Bilirubin	0.5	(0.4–0.7)	0.6	(0.4–0.7)	0.383

*ALBI grade* albumin–bilirubin grade, *ECOG PS* eastern cooperative oncology group performance status, *PD-L1* programmed cell death ligand-1, *ICI* immune-checkpoint inhibitor, *TPS* tumor proportion score.

^a^Adenocarcinoma versus all others, ^b^PD-L1 TPS ≥ 1% versus all others, ^c^Administered immune checkpoint inhibitors, Nivolumab versus all others.

### Association between ALBI grade and survival only in patients with superior PS

Since there was a correlation between inferior PS and ALBI grade 2b, 3 as shown in Table 2, we also analyzed the prognosis only in patients with PS 0 and 1 in the univariate Kaplan–Meier analysis. There were no differences in clinical variables between the ALBI grade 1, 2a and ALBI grade 2b, 3 groups, as shown in Table 3. Consequently, our results showed that the ALBI grade 1, 2a group had a significantly prolonged PFS (HR 0.66, 95% CI 0.43–0.99, *p* = 0.0425, Fig. 1c) and OS (HR 0.59, 95% CI 0.35–0.99, *p* = 0.0266, Fig. 1d), even in patients with PS 0–1.

**Table 3:** Association between ALBI Grade and clinical variables in patients with PS 0–1.

Characteristic	ALBI Grade 1, 2a		ALBI Grade 2b, 3		*p*
	(n = 86)		(n = 38)		
**Age, median—years (range)**	66	(36–88)	67	(44–80)	
< 70	54	(62.8)	26	(68.4)	0.684
≥ 70	32	(37.2)	12	(31.6)	
**Sex—no. (%)**					
Male	63	(73.3)	28	(73.7)	> 0.999
Female	23	(26.7)	10	(26.3)	
**Smoking history—no. (%)**					
Never	19	(22.1)	8	(21.1)	> 0.999
Current or former	67	(77.9)	30	(78.9)	
**Drinking history—no. (%)**					
Occasional / Never	49	(57.0)	25	(65.8)	0.429
Heavy / Moderate	37	(43.0)	13	(34.2)	
**ECOG PS—no. (%)**					
0–1	86	(100.0)	38	(100.0)	> 0.999
≥ 2	0	(0.0)	0	(0.0)	
**Common sites of metastasis—no. (%)**					
Brain	29	(33.7)	12	(31.6)	> 0.999
Bone	22	(25.6)	11	(28.9)	0.826
Lung	18	(20.9)	7	(18.4)	0.813
Liver	8	(9.3)	4	(10.5)	> 0.999
**Histology—no. (%)**					
Adenocarcinoma	58	(67.4)	25	(65.8)	> 0.999^a^
Squamous	22	(25.6)	9	(23.7)	
Other	6	(7.0)	4	(10.5)	
**PD-L1 status—no. (%)**					
TPS ≥ 50%	22	(25.6)	14	(36.8)	0.564^b^
TPS ≥ 1–49%	16	(18.6)	5	(13.2)	
TPS < 1%	24	(27.9)	6	(15.8)	
Not investigated	24	(27.9)	13	(34.2)	
**ICI line of treatment—no. (%)**					
First and second line	46	(53.5)	24	(63.2)	0.334
Third line or more	40	(46.5)	14	(36.8)	
**Administered ICIs—no, (%)**					
Nivolumab	46	(53.5)	20	(52.6)	> 0.999^c^
Pembrolizumab	20	(23.3)	13	(34.2)	
Pembrolizumab + chemotherapy	7	(8.1)	2	(5.3)	
Atezolizumab	13	(15.1)	3	(7.9)	
**Baseline steroid use—no, (%)**					
Yes	5	(5.8)	1	(2.6)	0.666
No	81	(94.2)	37	(97.4)	
**Laboratory tests—median, (IQR)**					
Albumin	3.9	(3.7–4.1)	3.0	(2.9–3.3)	< 0.001
Bilirubin	0.5	(0.4–0.7)	0.55	(0.4–0.7)	0.588

*ALBI grade* albumin–bilirubin grade, *ECOG PS* eastern cooperative oncology group performance status, *PD-L1* programmed cell death ligand-1, *ICI* immune-checkpoint inhibitor, *TPS* tumor proportion score, *IQR* interquartile range.

^a^Adenocarcinoma versus all others, ^b^PD-L1 TPS ≥ 1% versus all others, ^c^Administered immune checkpoint inhibitors, nivolumab versus all others.

### Prognostic significance of ALBI grade in multivariate analysis

Additionally, multivariate analysis for assessing survival outcome and patient characteristics was performed using the Cox proportional hazards model, as shown in Table 4. The variables such as age, sex, smoking history, drinking history, ECOG PS, ICI line of treatment, presence of liver metastasis, and ALBI grade have been included in multivariate analysis. Our results showed that pre-treatment ALBI grade, number of prior treatment regimens, and PS were independent prognostic factors for PFS. Pre-treatment ALBI grade, age at the start of treatment, and PS were independent prognostic factors for OS.

**Table 4:** Multivariate analysis of clinical variables associated with survival outcomes.

Characteristic	Progression-free survival			Overall survival		
	HR	95% CI	*p*	HR	95% CI	*p*
**Age (years)**						
< 70	0.73	0.49–1.08	0.111	0.61	0.39–0.96	0.031
≥ 70						
**Sex**						
Male	0.79	0.45–1.39	0.416	1.13	0.61–2.09	0.694
Female						
**Smoking history**						
Never	1.33	0.77–2.30	0.303	1.00	0.55–1.84	0.991
Current or former						
**Drinking history**						
Occasional/never	0.92	0.59–1.43	0.722	1.25	0.75–2.07	0.388
Heavy/moderate						
**ECOG PS**						
0–1	0.35	0.19–0.64	< 0.001	0.24	0.13–0.45	< 0.001
≥ 2						
**ICI line of treatment**						
First and second line	0.65	0.44–0.97	0.033	0.73	0.47–1.14	0.173
Third line or more						
**Liver metastasis**						
No	0.98	0.51–1.87	0.951	1.29	0.63–2.65	0.485
Yes						
**ALBI grade**						
Grade 1, Grade 2a	0.57	0.38–0.86	0.007	0.45	0.29–0.72	0.001
Grade 2b, Grade 3						

*HR* hazard ratio, *CI* confidence interval, *ECOG PS* eastern cooperative oncology group performance status, *ALBI grade* albumin–bilirubin grade, *ICI* immune-checkpoint inhibitor.

### ALBI grade and survival in propensity score matching analysis

**Figure 2:**
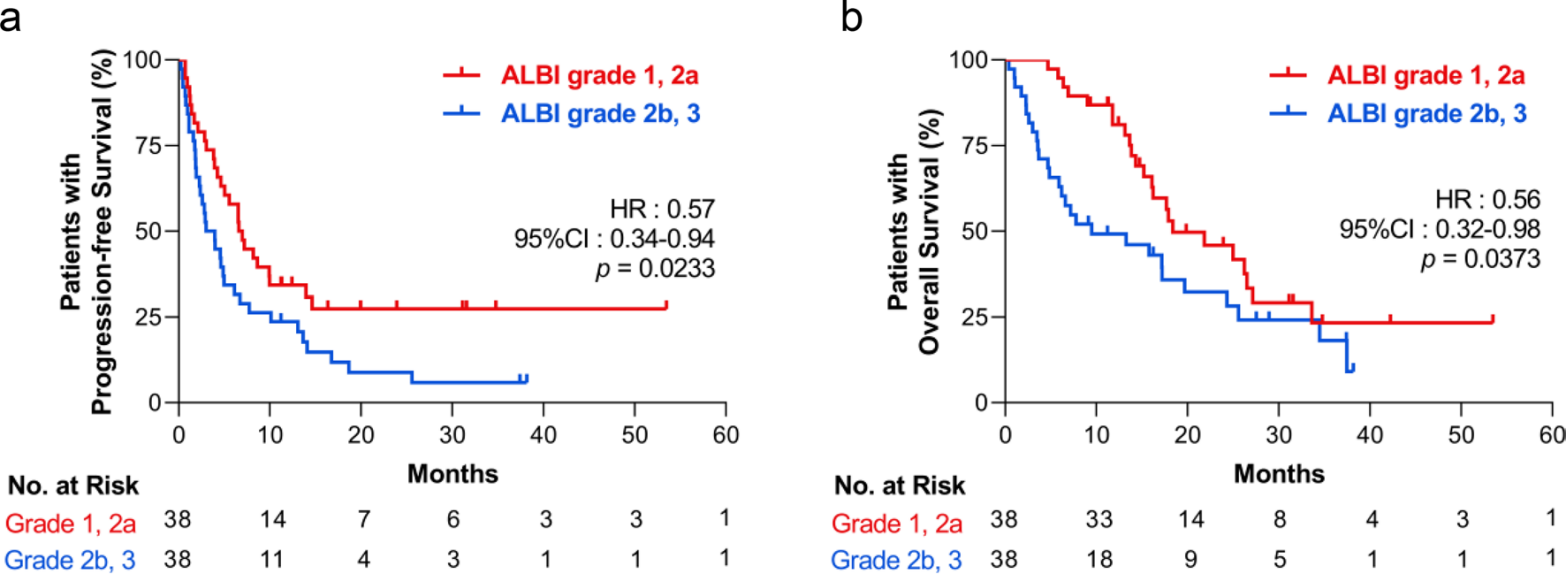
Kaplan–Meier survival analysis according to pre-treatment ALBI grade in patients who were selected by propensity score matching. (**a**) Progression-free survival and (**b**) overall survival. (N = 76). ALBI, albumin–bilirubin; HR, hazard ratio; CI, confidence interval.

To further validate the impact of ALBI grade on survival results in treatment with ICI, we employed a propensity score matching analysis to equalize the patient background information. As a result of score matching, we extracted 38 paired patients from the ALBI grade 1, 2a, and grade 2b, 3 groups. The violin plot of the propensity score before and after matching has been described in Supplementary Fig. 3a–c. There were no differences in patients’ characteristics among the extracted 38-paired patients, as summarized in Table 5. Standardized mean difference between ALBI grade 1, 2a, and grade 2b, 3 groups were also confirmed before and after the propensity score matching (Supplementary Fig. 3d). Each laboratory data profile in both groups differed slightly from the analysis involving all patients (Supplementary Fig. 4a–c). Finally, in a univariate Kaplan–Meier analysis, the ALBI grade 1, 2a group had a significantly longer PFS (HR 0.57, 95% CI 0.34–0.94, *p* = 0.0233, Fig. 2a) and OS (HR 0.56, 95% CI 0.32–0.98, *p* = 0.0373, Fig. 2b) than the ALBI grade 2b, 3 group. Therefore, a superior ALBI grade at pre-treatment indicates prolonged PFS and OS in patients with NSCLC treated with ICIs.

**Table 5:** Association between ALBI Grade and clinical variables in the propensity score-matched group.

Characteristic	ALBI Grade 1, 2a		ALBI Grade 2b, 3		*p*
	(n = 38)		(n = 38)		
**Age, median—years (range)**	66	(38—88)	67	(44—84)	
< 70	26	(68.4)	25	(65.8)	> 0.999
≥ 70	12	(31.6)	13	(34.2)	
**Sex—no. (%)**					
Male	29	(76.3)	29	(76.3)	> 0.999
Female	9	(23.7)	9	(23.7)	
**Smoking history—no. (%)**					
Never	9	(23.7)	7	(18.4)	0.779
Current of former	29	(76.3)	31	(81.6)	
**Drinking history—no. (%)**					
Occasional/never	26	(68.4)	24	(63.2)	0.809
Heavy/moderate	12	(31.6)	14	(36.8)	
**ECOG PS—no. (%)**					
0–1	37	(97.4)	37	(97.4)	> 0.999
≥ 2	1	(2.6)	1	(2.6)	
**Common sites of metastasis—no. (%)**					
Brain	15	(39.5)	12	(31.6)	0.632
Bone	13	(34.2)	16	(42.1)	0.637
Lung	7	(18.4)	7	(18.4)	> 0.999
Liver	2	(5.3)	3	(7.9)	> 0.999
**Histology—no. (%)**					
Adenocarcinoma	26	(68.4)	24	(63.2)	0.809^a^
Squamous	8	(21.1)	10	(26.3)	
Other	4	(10.5)	4	(10.5)	
**PD-L1 status—no. (%)**					
TPS ≥ 50%	9	(23.7)	14	(36.8)	0.818^b^
TPS 1–49%	13	(34.2)	6	(15.8)	
TPS < 1%	9	(23.7)	6	(15.8)	
Not investigated	7	(18.4)	12	(31.6)	
**ICI line of treatment—no. (%)**					
First and second line	23	(60.5)	24	(63.2)	> 0.999
Third line or more	15	(39.5)	14	(36.8)	
**Administered ICIs—no, (%)**					
Nivolumab	21	(55.2)	20	(52.6)	> 0.999^c^
Pembrolizumab	9	(23.7)	13	(34.2)	
Pembrolizumab + chemotherapy	3	(7.9)	2	(5.3)	
Atezolizumab	5	(13.2)	3	(7.9)	
**Baseline steroid use—no, (%)**					
Yes	3	(7.9)	1	(2.6)	0.615
No	35	(92.1)	37	(97.4)	
**Laboratory tests—median, (IQR)**					
Albumin	3.95	(3.8–4.1)	3.0	(2.9–3.3)	< 0.001
Bilirubin	0.5	(0.4–0.7)	0.5	(0.4–0.7)	0.836

*ALBI grade* albumin–bilirubin grade, *ECOG PS* eastern cooperative oncology group performance status, *PD-L1* programmed cell death ligand-1, *ICI* immune-checkpoint inhibitor, *TPS* tumor proportion score, *IQR* interquartile range.

^a^Adenocarcinoma versus all others, ^b^PD-L1 TPS ≥ 1% versus all others, ^c^Administered of immune checkpoint inhibitors, Nivolumab versus all others.

### Comparison of ALBI grade with related biomarkers for predicting survival

Recently, mGPS, PNI, and NLR have been reported as prognostic biomarkers for systemic nutrition and immune condition in treatment with ICI^16–18^. We performed a ROC analysis to compare the prognostication ability of these markers. The results are summarized in Supplementary Table 1. The ALBI score showed an equivalent or much better AUC than the other markers.

### Association of ALBI grade and irAE onsets

Of the 140 patients, 69 (49.3%) patients had any grade of irAEs, and 15 patients (10.7%) experienced grade 3 or higher ones during the observation period. Twenty-two patients (15.7%) had more than two irAEs, and we observed 93 irAEs in total. Detailed information on irAE classification, incidence rate, severity is summarized in Supplementary Table 2. Common irAEs were skin toxicity (26 cases), pneumonitis (19 cases), hypothyroidism or thyroiditis (10 cases), hepatitis (8 cases), and adrenal insufficiency or hypophysitis (6 cases). There was no significant difference in pre-treatment ALBI score between the patients with or without the development of any grade irAE (Supplementary Fig. 5a). Incidence rate of each grade irAE in ALBI grade 1, 2a group (grade 1, 14.8%; grade 2, 21.6%; grade 3 or more 12.5%) and grade 2b, 3 group (grade 1, 15.4%; grade 2, 26.9%; grade 3 or more 7.7%) was similar to each other (Supplementary Fig. 5b).

## Discussion

In this study, we retrospectively investigated the impact of pre-treatment hepatic impairment, as measured by the ALBI score, on the prognosis and safety of ICIs in NSCLC. This is the first study to incorporate the ALBI grade system into assessing liver function and showing the importance of pre-treatment hepatic reserve in patients with NSCLC who received ICI therapy. We found that a superior ALBI grade was a significant prognostic factor in the univariate, multivariate, and propensity score matching analyses. These results indicate that the pre-treatment liver function has a remarkable effect on ICI therapy in NSCLC. However, there was no association between irAE onset and ALBI grade.

In this investigation, we used the ALBI score to evaluate liver function. Currently, the Child–Pugh score is the most important scoring system. However, it is not suitable for assessing liver function in patients with cancer because most studies on HCC include even patients with Child–Pugh A^9^. The ALBI score was developed to predict the prognosis of HCC; it was originally defined by the following classification of mortality risk in patients with HCC from various etiologies: Grade 1 for 25% of low-risk, grade 2 for 65% of moderate-risk, and grade 3 for the remaining 10% of high-risk^9–12^. According to our dataset for NSCLC, 37.1%, 57.9%, and 5.0% of patients were classified as ALBI grade 1, grade 2, and grade 3, respectively. Although there were slightly more patients with a better liver function than the definition of HCC, the proportions were mostly consistent, and we considered that the application of ALBI grade to our investigation in NSCLC was reasonable. To date, Pinato and Kaneko et al. have considered the prognostic ability of ALBI grade in HCC treated with immunotherapy^19^. They showed that the pre-treatment ALBI grade predicted prolonged OS, and that it was superior to the Child–Pugh grade in predicting mortality. In addition, kinoshita et al. have compared pre-operative ALBI grade with clinicopathological characteristics and prognosis in patients with resectable non-small cell lung cancer, and reported that ALBI grade 2, 3 was associated with poor prognosis^20^. These previous reports support our results regarding its potential as a suitable biomarker for assessing prognosis in NSCLC.

The physiological effects of impaired liver function on the systemic immune response have been studied in alcoholic liver injury and viral hepatitis^5,6^. Previous studies indicated that decreased liver function causes changes in T cell repertoires, and its effect has been evident since the early stage of cirrhosis, regardless of the disease etiology^6,7^. In detail, the immune checkpoint molecules: T cell immunoglobulin and mucin domain 3 (TIM-3), cytotoxic T-lymphocyte-associated protein 4 (CTLA-4), and PD-1 expressed on activated cytotoxic T cells are upregulated in patients with cirrhosis^21^. There was no correlation between the liver function assessed using the ALBI Grade and alcohol consumption or liver metastasis in this present study. Therefore, the etiology of hepatic impairment is unclear. Although further studies are needed, impaired liver function and subsequent changes in the immune microenvironment may be involved in the tumor escape mechanism from systemic immune function.

Furthermore, we have limited information on how hepatic impairment affects the pharmacokinetics of antibody drugs^22^. In previous studies, trastuzumab, another antibody drug, has been shown to increase drug clearance in patients with hypoalbuminemia^23^. It has been suggested that this mechanism may be due to hyperglobulinemia and hypoalbuminemia associated with hepatic impairment. Antibody drugs have a long half-life because they can escape proteolysis by lysosomes through binding with neonatal Fc receptors (FcRn), an endogenous IgG recycling mechanism^24,25^. The increased endogenous IgG can result in competitive FcRn binding with therapeutic antibody drugs, increased clearance of the drug, and decreased exposure of the target^26^. Additionally, our data showed a reduction in the albumin-globulin ratio in the hepatic impairment group, and its influence on antibody drug clearance was expected.

Moreover, irAEs are immune-mediated side effects that develop in various organs following treatment with ICIs^27^. We confirmed that there was a similar incidence of each grade irAEs in both groups dichotomized by pre-treatment ALBI score. As shown in a previous study, increased levels of immune checkpoint molecules, which negatively regulate the function of immune cells, have been observed in patients with cirrhosis. Based on these reports, it is assumed that an impaired hepatic reserve promotes the tumor tolerance to immune surveillance and is not directly related to the accelerated activation of immune function, which is related to the development of irAEs.

Several routine biomarkers are available for predicting the therapeutic efficacy of ICIs^28–30^. For instance, NLR as systemic inflammation and immune marker, GPS and PNI as nutrition status markers, as well as cachexia and sarcopenia, have been reported as prognostic biomarkers^16–18,31–33^. However, we need to consider that many of these biomarkers correlate with the patient's age and PS. Herein, we included only patients with PS 0 or 1 in the univariate survival analysis; we found that a better ALBI grade predicted a better prognosis, regardless of any difference in age or patient background. These results indicate that pre-treatment ALBI grade is a better prognostic biomarker than those used currently. Even in patients with similar activities of daily living and physical states at the start of treatment, they could have different clinical outcomes, depending on the hepatic reserve. Conversely, the most impressive feature of ICI regimens different from conventional chemotherapy and molecular targeted drugs is the increase in patients with long-term durable survival^34,35^. Although ALBI grade is a prognostic factor throughout ICI treatment, it is still insufficient to accurately predict patients with ICI-specific long-term prognosis. Pursuing biomarkers that can distinguish patients with a long-term prognosis will be an important goal for the further advancement of ICI therapy.

There are several limitations to this study. First, this was a single-center, retrospective analysis. We carefully investigated the patient's background characteristics, which may have affected the ALBI grade and prognosis. However, the confounding factors that could not be assessed in this study may have distorted our results because there was no randomization. Since the multivariate analysis was performed using a large number of covariates in a limited patient population, the reliability of this analysis also needs to be confirmed with a further large population analysis. Furthermore, the current optimal regimen for NSCLC without targetable driver alteration is selected due to tumor cell PD-L1 expression and histology^36^. We need further clinical studies with more subdivided populations to establish the influence of pre-treatment ALBI grade on ICI treatment.

In conclusion, our study showed that the pre-treatment hepatic reserve using the ALBI grade could remarkably predict survival in patients with NSCLC treated with ICI. Our results suggest that ALBI grade could be an essential biomarker for predicting the efficacy of treatment with ICI. Throughout the establishment of these biomarkers, we further aim to identify patients in whom ICI therapy is beneficial and to prolong the survival of these patients.

## Supplementary Information

Supplementary Information.

## Data Availability

The data that support the findings of this study are available from the corresponding authors upon reasonable request.
