# Supplementary material for: Prognostic significance of pre-treatment ALBI grade in advanced non-small cell lung cancer receiving immune checkpoint therapy
Source: Sci Rep. 2021 Jul 23;11:15057. doi: 10.1038/s41598-021-94336-9 (PMC8302741; doi:10.1038/s41598-021-94336-9)
Supplement: Supplementary file 1 — Supplementary Information. [file 41598_2021_94336_MOESM1_ESM.docx]

Prognostic significance of pre-treatment hepatic function in advanced non-small cell lung cancer receiving immune checkpoint therapy

Ryosuke Matsukane^a,b^, Hiroyuki Watanabe^a^, Kojiro Hata^a,b^, Kimitaka Suetsugu^a^, Toshikazu Tsuji^a^, Nobuaki Egashira^a,b^, Yoichi Nakanishi^c^, Isamu Okamoto^c^, Ichiro Ieiri^a,b*^

^a^ Department of Pharmacy, Kyushu University Hospital, Fukuoka, Japan

^b^ Department of Clinical Pharmacology and Biopharmaceutics, Graduate School of Pharmaceutical Sciences, Kyushu University, Fukuoka, Japan

^c^Research Institute for Diseases of the Chest, Graduate School of Medical Sciences, Kyushu University, Fukuoka, Japan

Ryosuke Matsukane: matsukane.ryosuke.096@m.kyushu-u.ac.jp

Hiroyuki Watanabe: watanabe.hiroyuki.407@m.kyushu-u.ac.jp

Kojiro Hata: hata.kojiro.151@m.kyushu-u.ac.jp

Kimitaka Suetsugu: suetsugu.kimitaka.345@m.kyushu-u.ac.jp

Toshikazu Tsuji: tsuji.toshikazu.123@m.kyushu-u.ac.jp

Nobuaki Egashira: n-egashi@pharm.med.kyushu-u.ac.jp

Yoichi Nakanishi: naka24@e-mail.jp

Isamu Okamoto: okamoto.isamu.290@m.kyushu-u.ac.jp

*Correspondence to:

Ichiro Ieiri, PhD

Department of Pharmacy, Kyushu University Hospital. Department of Clinical Pharmacology and Biopharmaceutics, Graduate School of Pharmaceutical Sciences, Kyushu University, 3-1-1 Maidashi, Higashi-ku, Fukuoka 812-8582, Japan

Tel: +81-92-642-5918, Fax: +81-92-642-5937

E-mail: ieiri@phar.kyushu-u.ac.jp

ORCID

Ryosuke Matsukane: 0000-0001-5444-2443

Nobuaki Egashira: 0000-0002-3673-6824

Ichiro Ieiri: 0000-0002-3543-2562

| **Supplementary Table 1. ROC analysis in various biomarkers for predicting PFS and OS** | | | | | | | | |
| --- | --- | --- | --- | --- | --- | --- | --- | --- |
|  | 6-month PFS | | | | 6-month OS | | | |
| predictors | AUC | 95% CI | *p* | cut-off value | AUC | 95% CI | *p* | cut-off value |
| ALBI score | 0.60 | 0.50 - 0.69 | 0.049 | -2.10 | 0.74 | 0.64 - 0.84 | < 0.0001 | -2.22 |
| mGPS | 0.57 | 0.47 - 0.66 | 0.174 |  | 0.74 | 0.64 - 0.83 | < 0.0001 | 1.5 |
| PNI | 0.61 | 0.51 - 0.70 | 0.033 | 41.1 | 0.75 | 0.65 - 0.85 | < 0.0001 | 40.1 |
| NLR | 0.54 | 0.45 - 0.64 | 0.401 |  | 0.74 | 0.64 - 0.84 | < 0.0001 | 5.5 |

PFS: progression-free survival, OS: overall survival, ROC: receiver operating characteristic, AUC: area under the curve, CI: confidence interval, ALBI score: albumin-bilirubin score, mGPS: modified Glasgow prognostic score, PNI: prognostic nutritional index, NLR: neutrophil to lymphocyte ratio

**Supplementary Table 2. Detail of immune-related adverse events**

|  |  | Grading, n (%) | | | | | |
| --- | --- | --- | --- | --- | --- | --- | --- |
|  | n | 1 | | 2 | | ≥3 | |
| **Total** | 93 | 40 | (43.0) | 37 | (39.8) | 16 | (17.2) |
|  |  |  |  |  |  |  |  |
| **irAE subtype** |  |  |  |  |  |  |  |
| skin toxicity | 28 | 16 | (57.1) | 10 | (35.7) | 2 | (7.1) |
| pneumonitis | 19 | 11 | (57.9) | 2 | (10.5) | 6 | (31.6) |
| hypothyroidism, thyroiditis | 10 | 2 | (20.0) | 8 | (80.0) |  | (0.0) |
| adrenal insufficiency, hypophysitis | 6 | 0 | (0.0) | 5 | (83.3) | 1 | (16.7) |
| type 1 diabetes | 0 | 0 | – | 0 | – | 0 | – |
| colitis | 6 | 0 | (0.0) | 4 | (66.7) | 2 | (33.3) |
| hepatitis | 8 | 4 | (50.0) | 2 | (25.0) | 2 | (25.0) |
| nephritis | 2 | 0 | (0.0) | 2 | (100.0) | 0 | (0.0) |
| musculoskeletal toxicity | 3 | 1 | (33.3) | 1 | (33.3) | 1 | (33.3) |
| nervous system toxicity | 2 | 1 | (50.0) | 0 | (0.0) | 1 | (50.0) |
| cardiovascular toxicity | 1 | 1 | (100.0) | 0 | (0.0) | 0 | (0.0) |
| ocular toxicity | 1 | 1 | (100.0) | 0 | (0.0) | 0 | (0.0) |
| hematologic toxicity | 1 | 0 | (0.0) | 1 | (100.0) | 0 | (0.0) |
| other | 6 | 3 | (50.0) | 2 | (33.3) | 1 | (16.7) |

irAE: immune-related adverse events

**
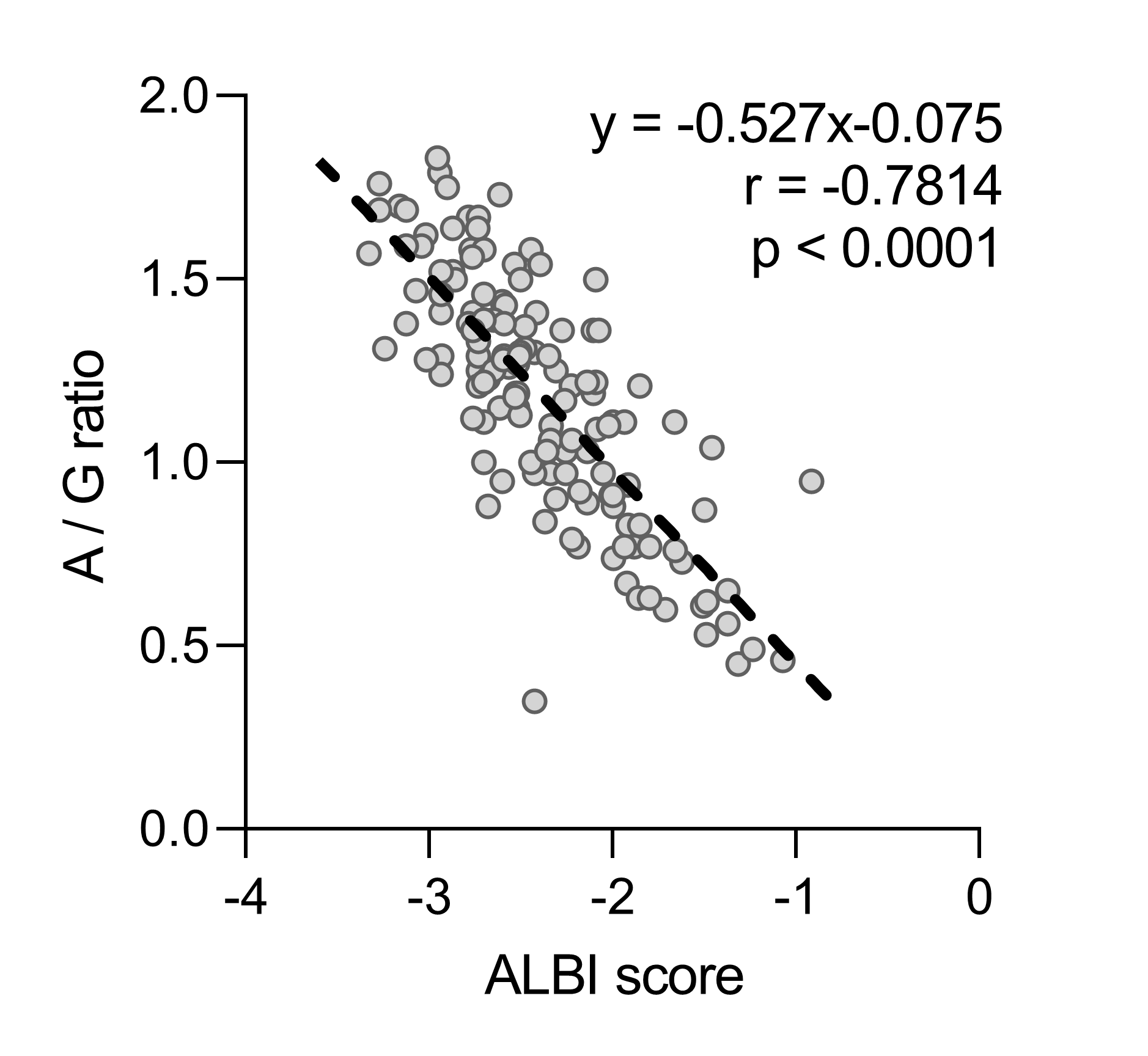
Supplementary Fig. 1**

Supplementary Fig. 1

Correlation between albumin-bilirubin (ALBI) score and albumin-globulin (A/G) ratio. Statistical analysis was performed using the Pearson’s correlation coefficient analysis.

**Supplementary Fig. 2**


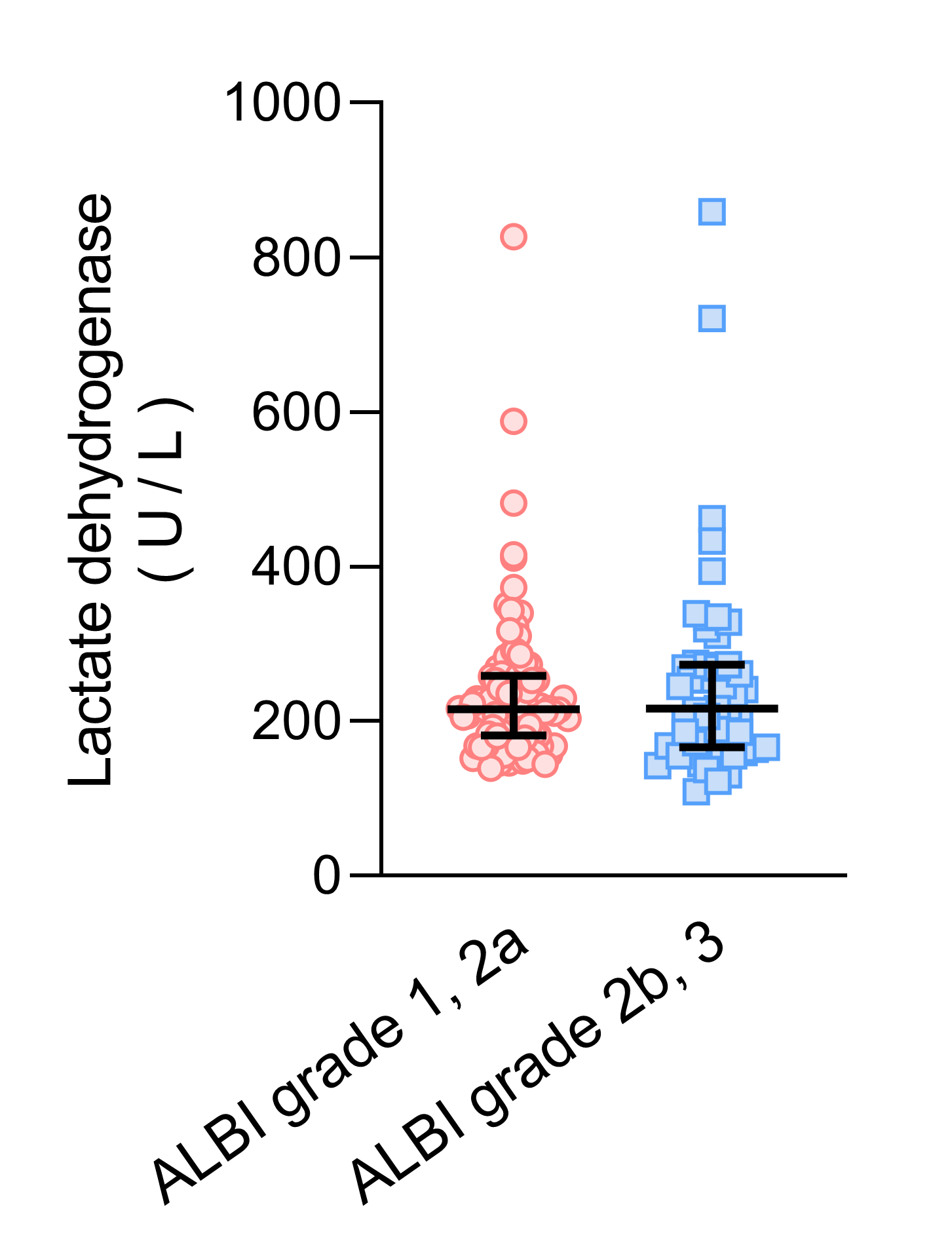

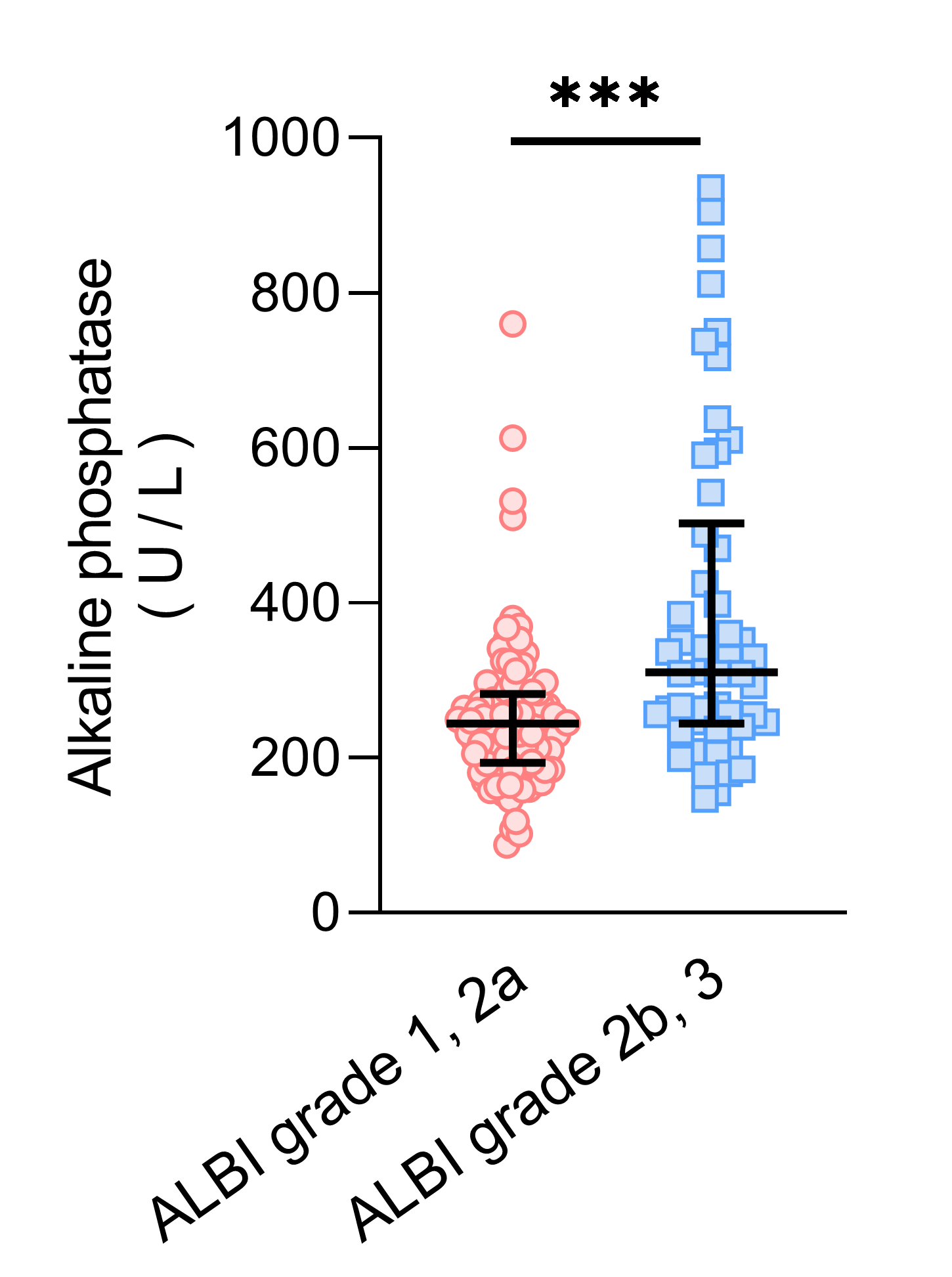

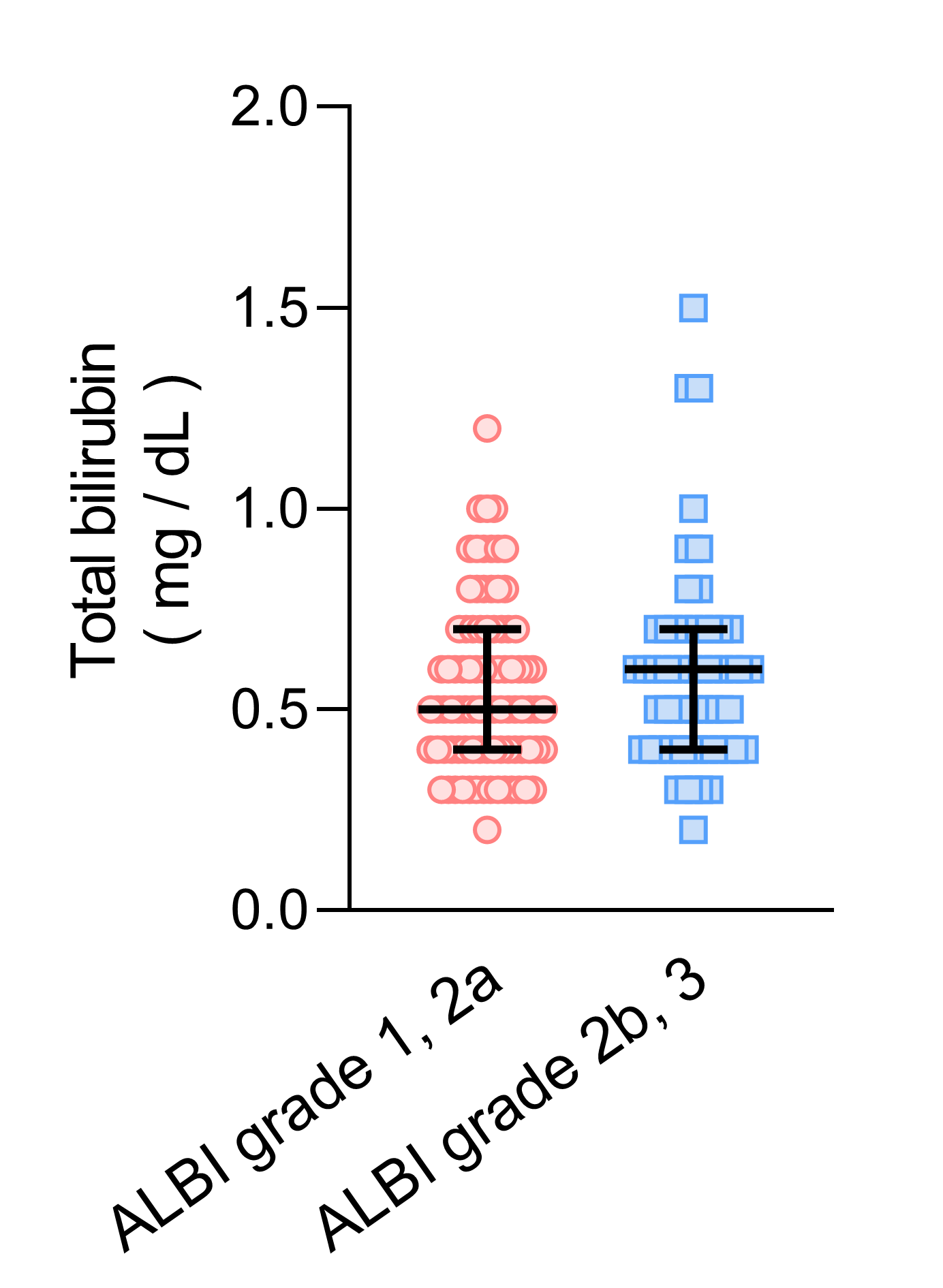

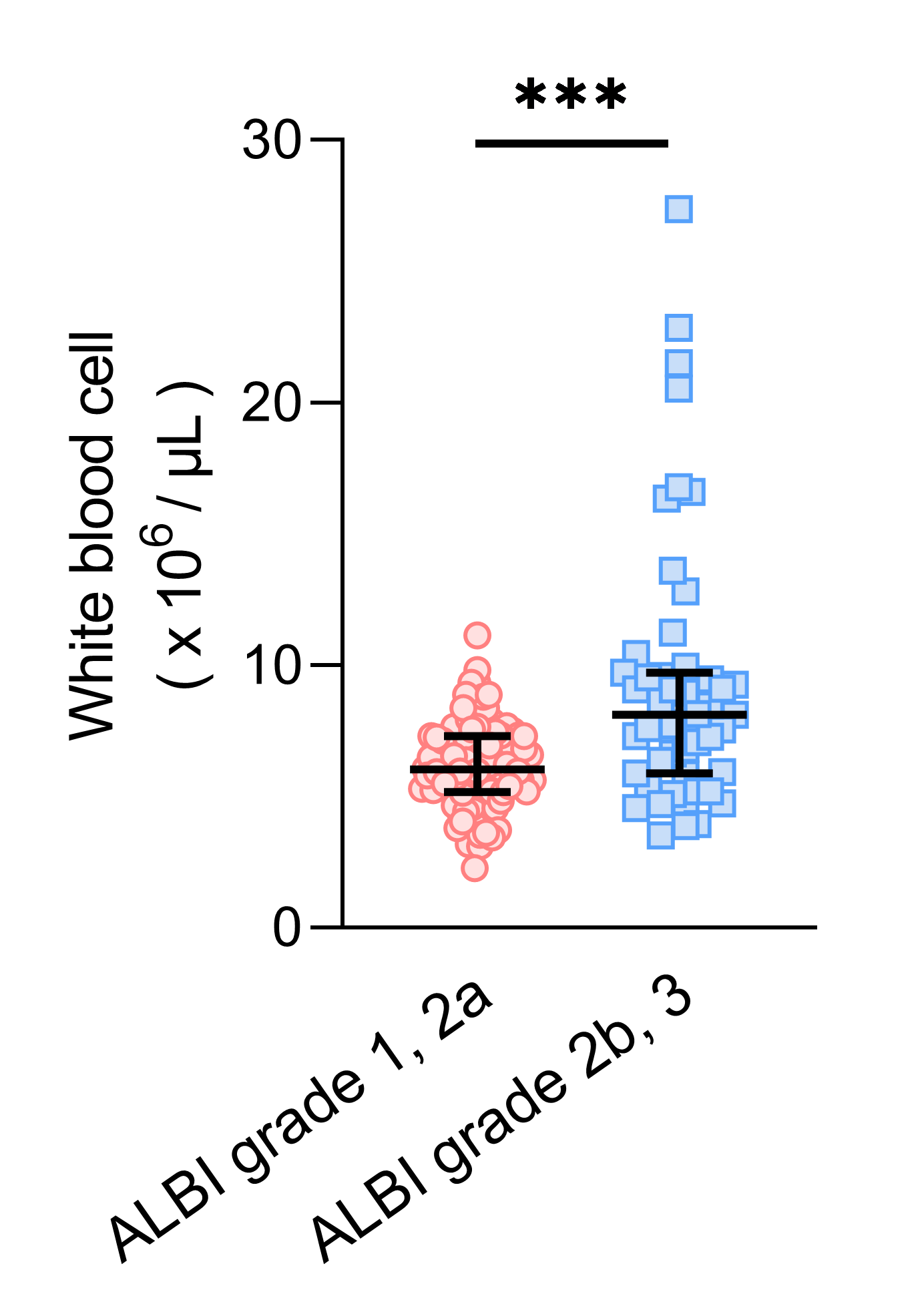

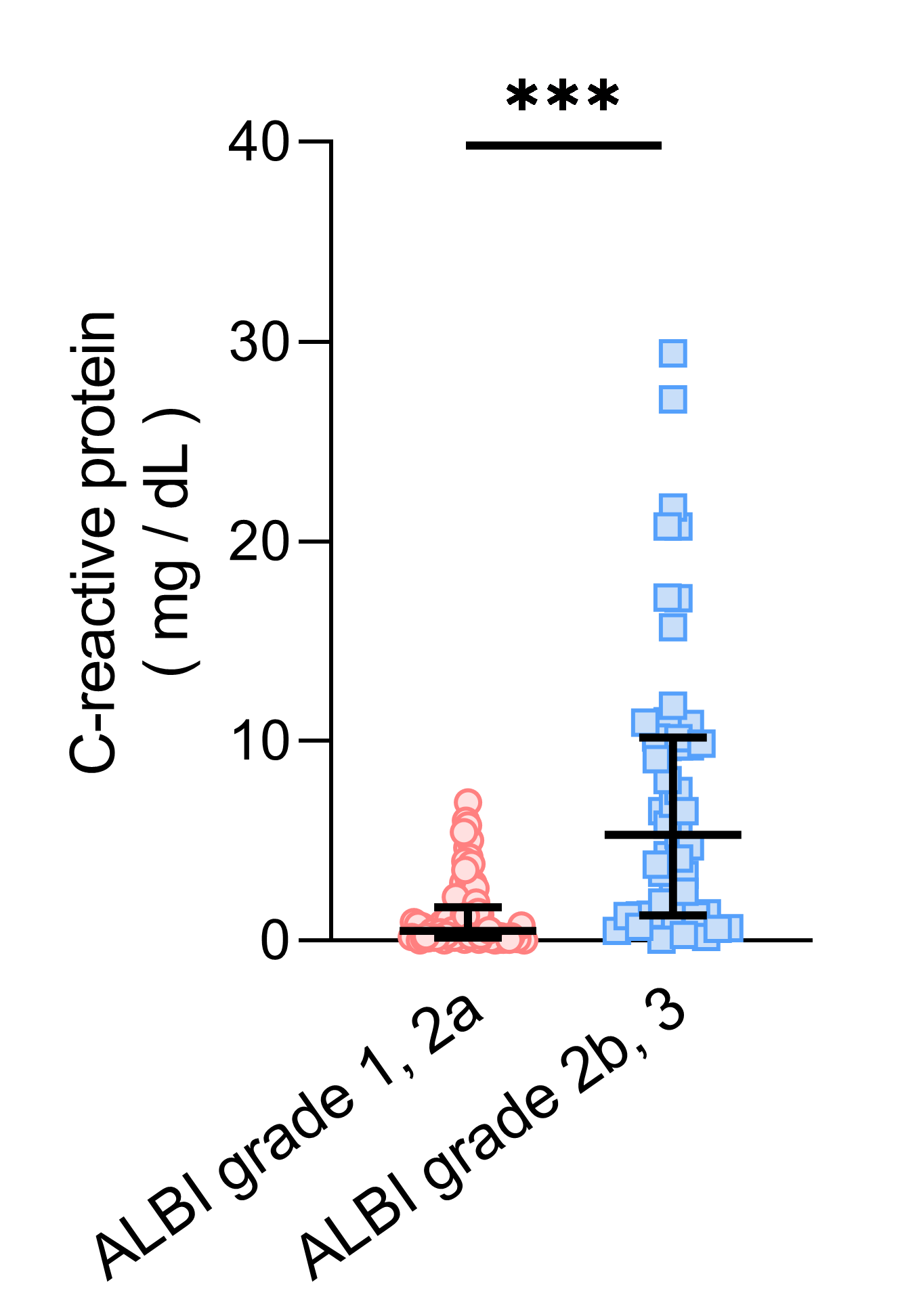

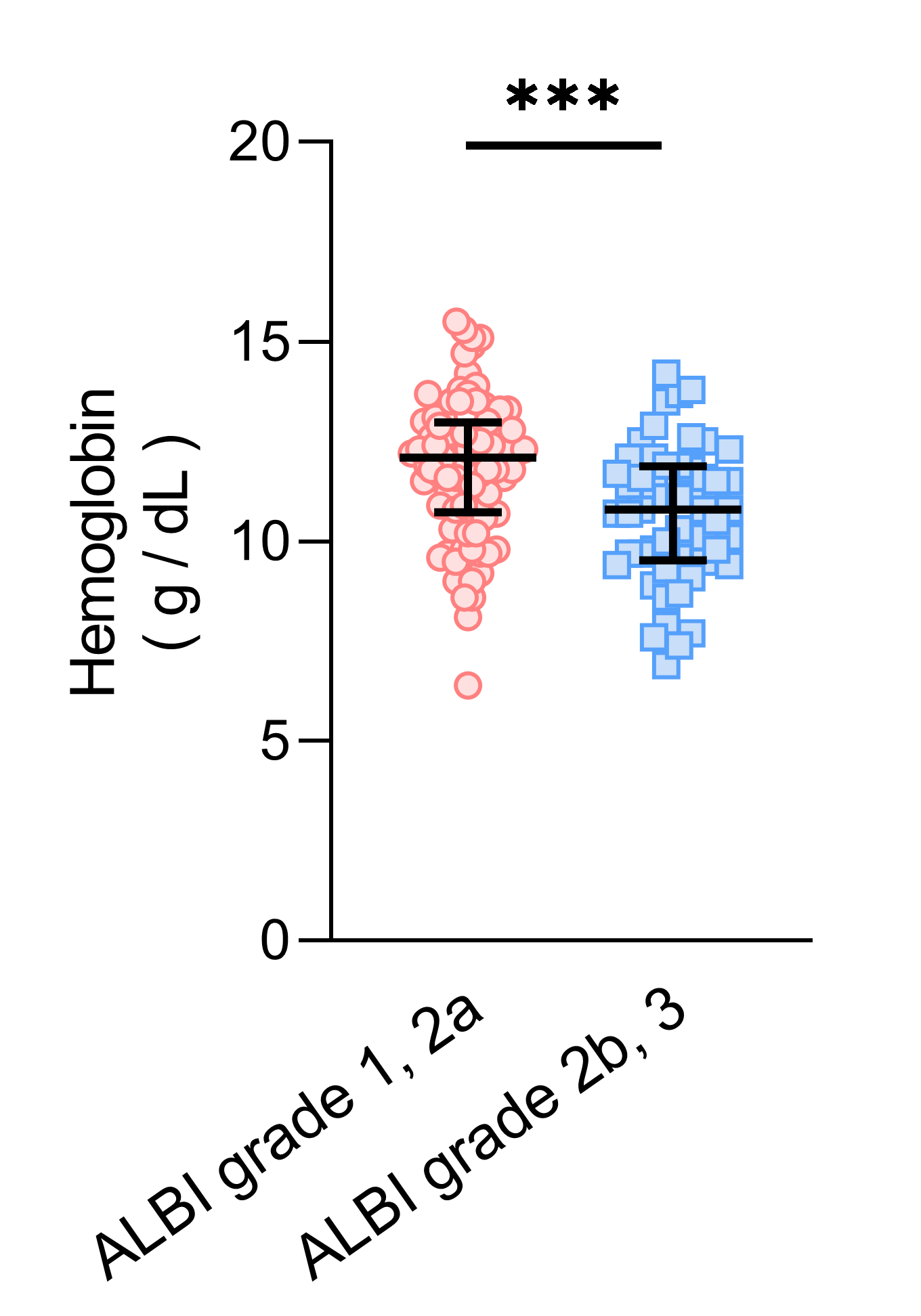

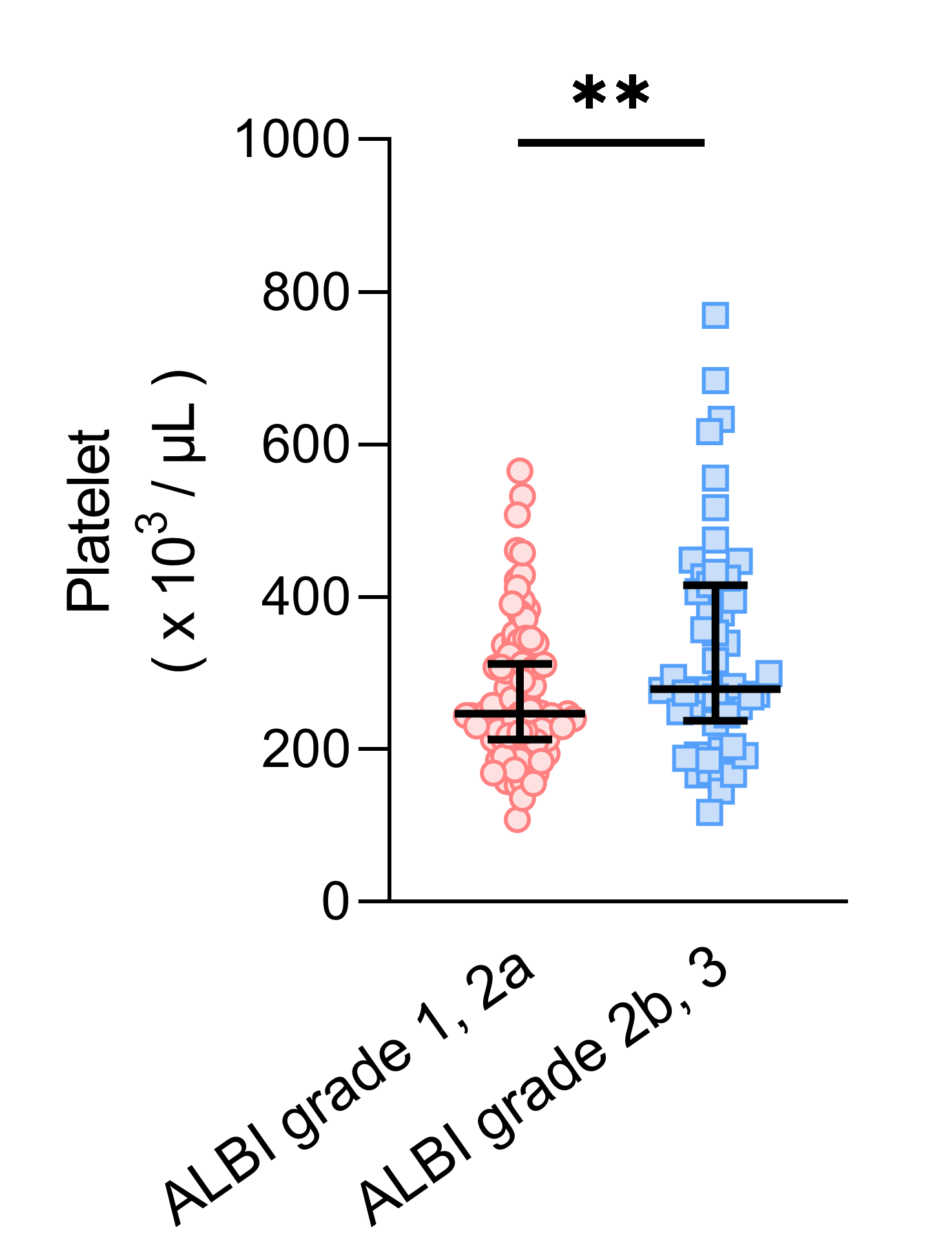

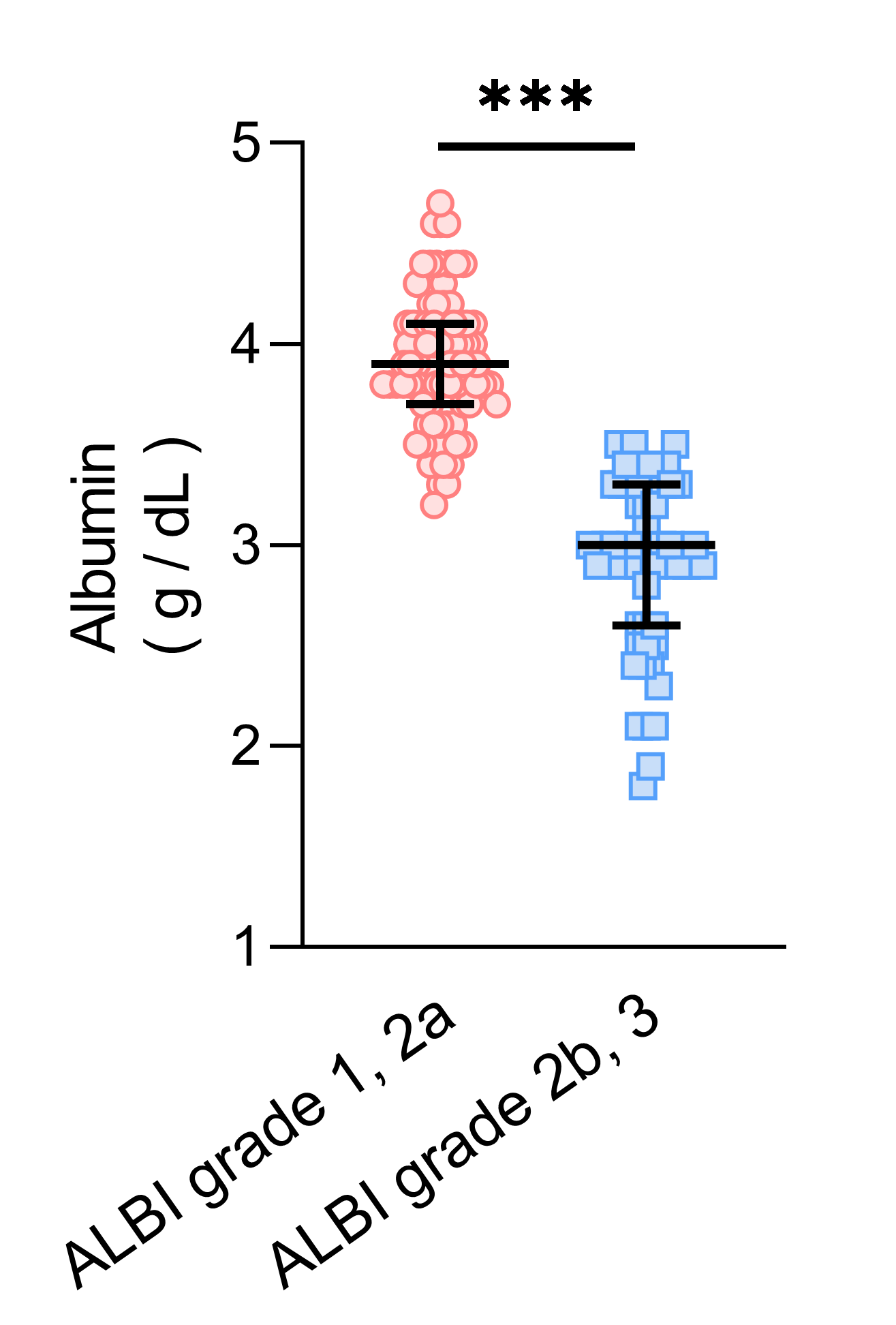

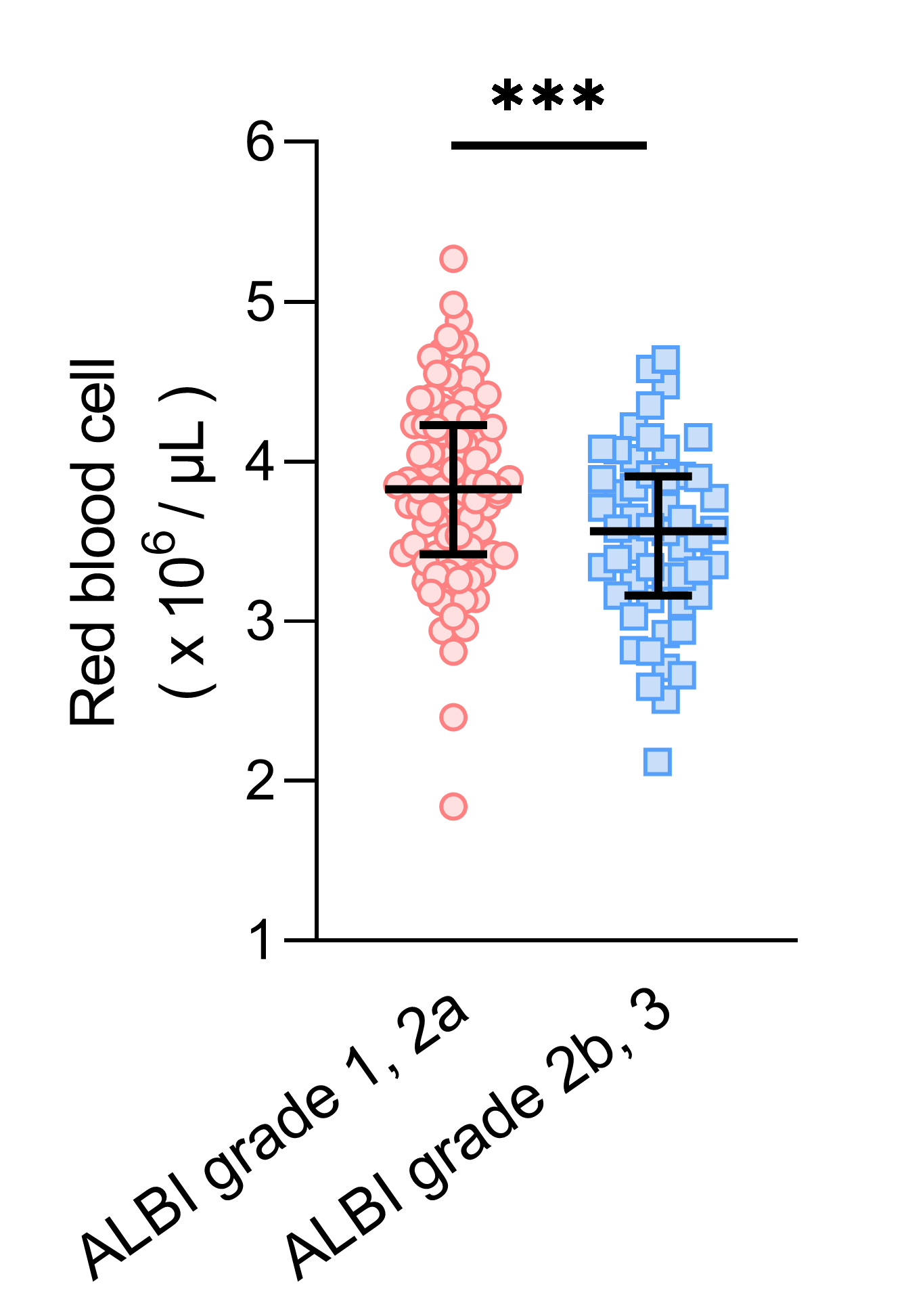

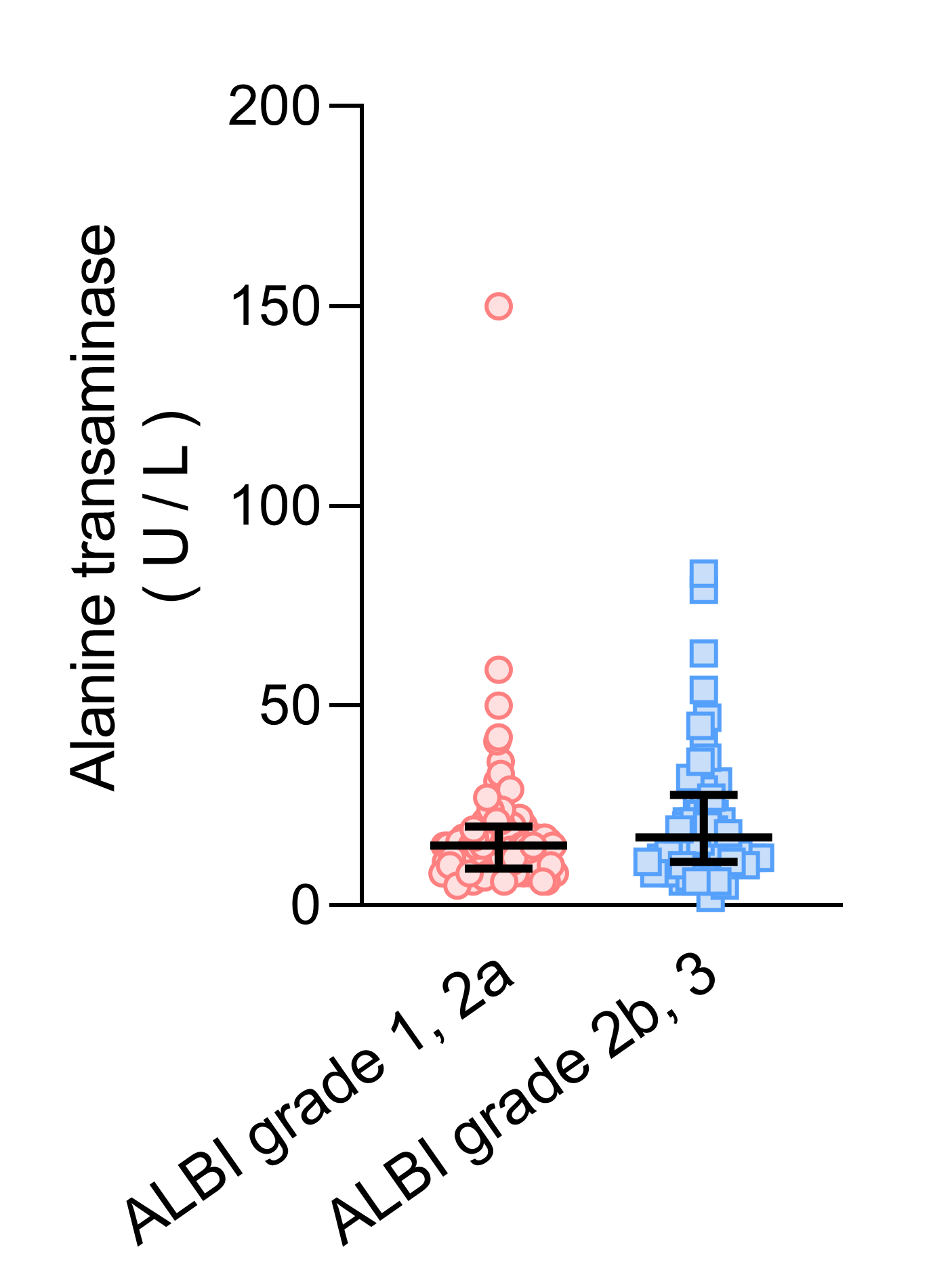

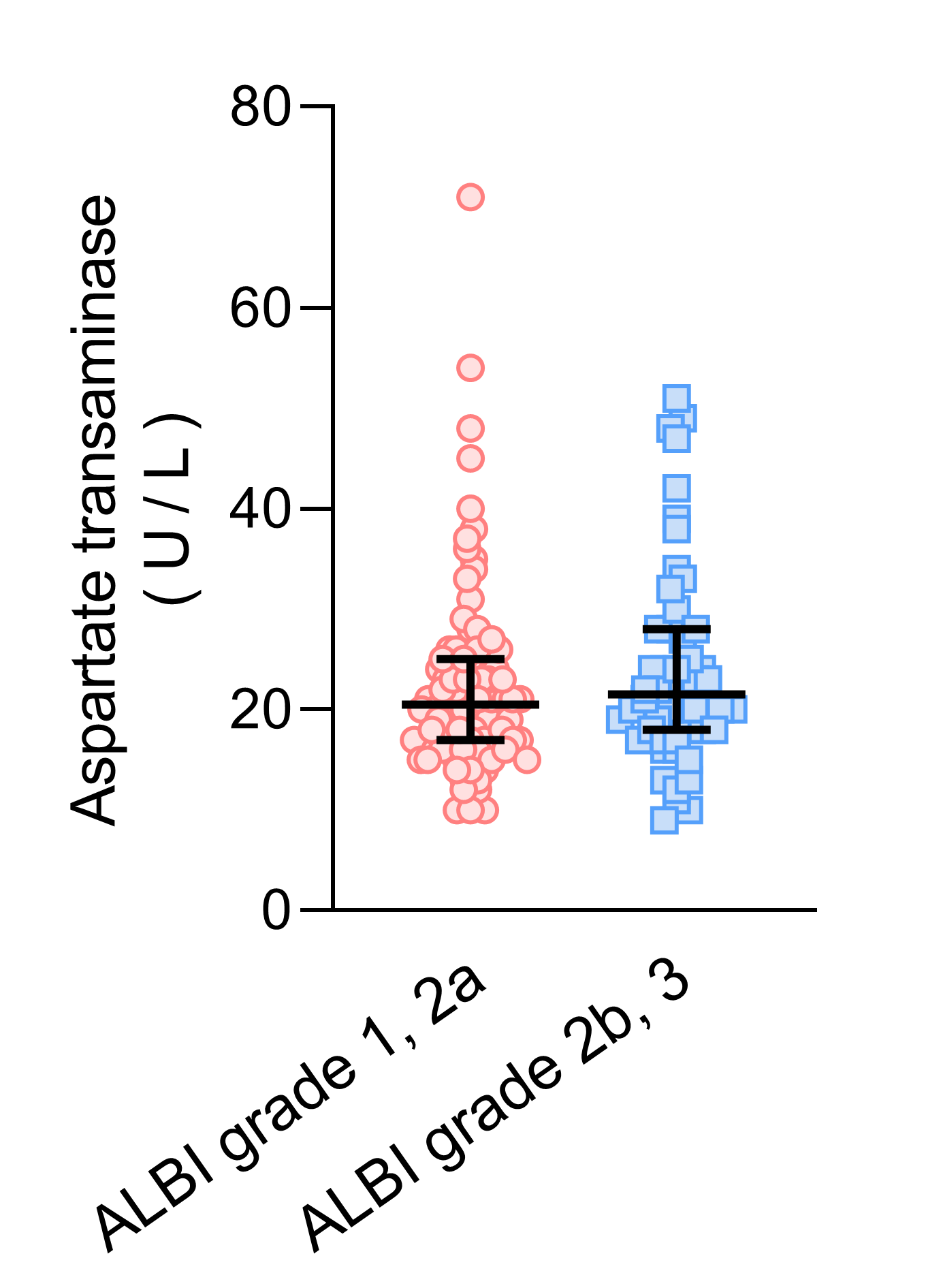


c

a

b

Supplementary Fig. 2

Pretreatment laboratory data of patients with non-small cell lung cancer receiving immune checkpoint inhibitors, according to albumin-bilirubin (ALBI) grade dichotomization. The ALBI grade 1, 2a group and ALBI grade 2b, 3 group included 88 and 52 patients, respectively. All data are shown as median with interquartile range. Statistical analysis was performed using the Mann-Whitney U test. ** p < 0.01, *** p < 0.005

**Supplementary Fig. 3**


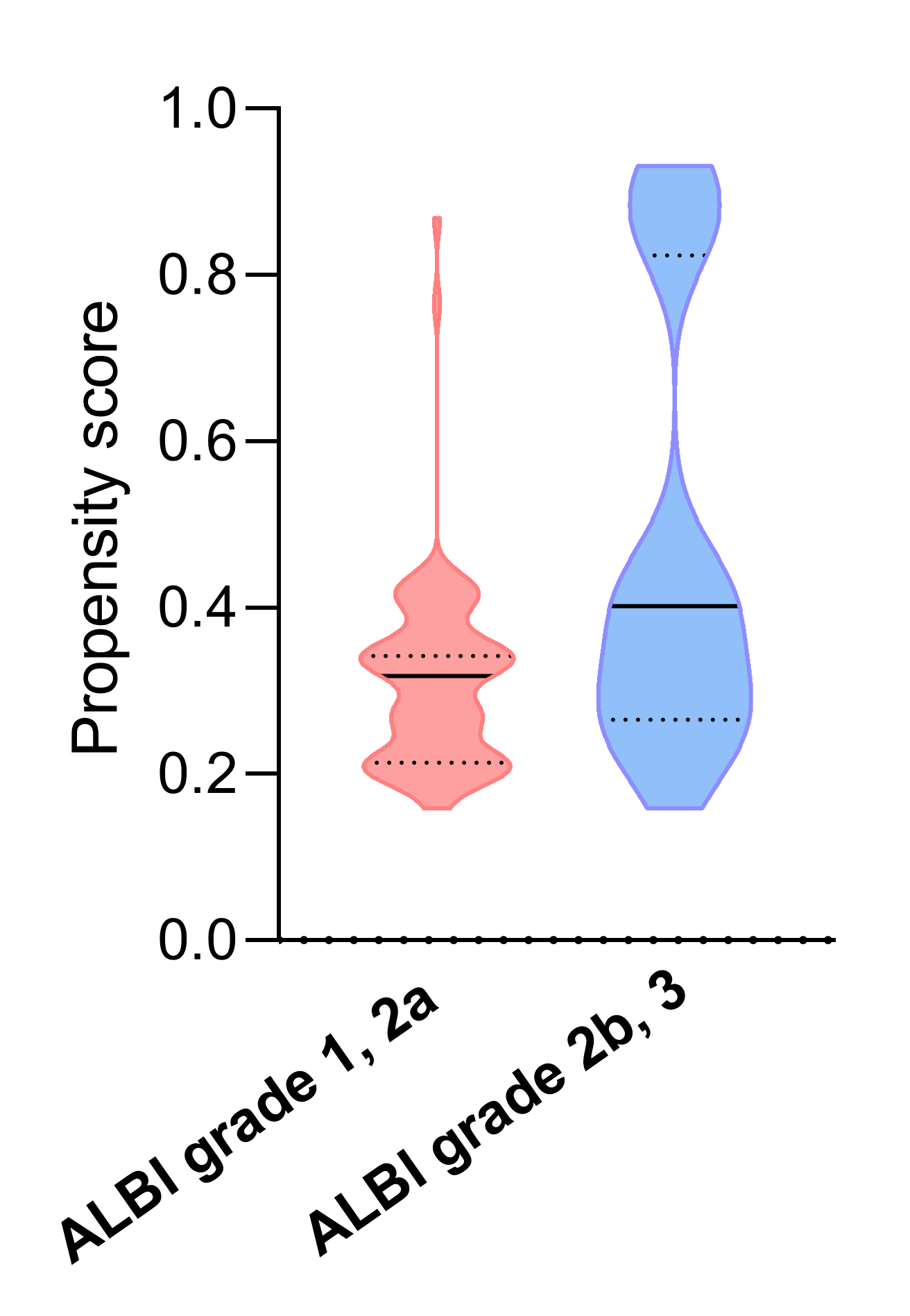

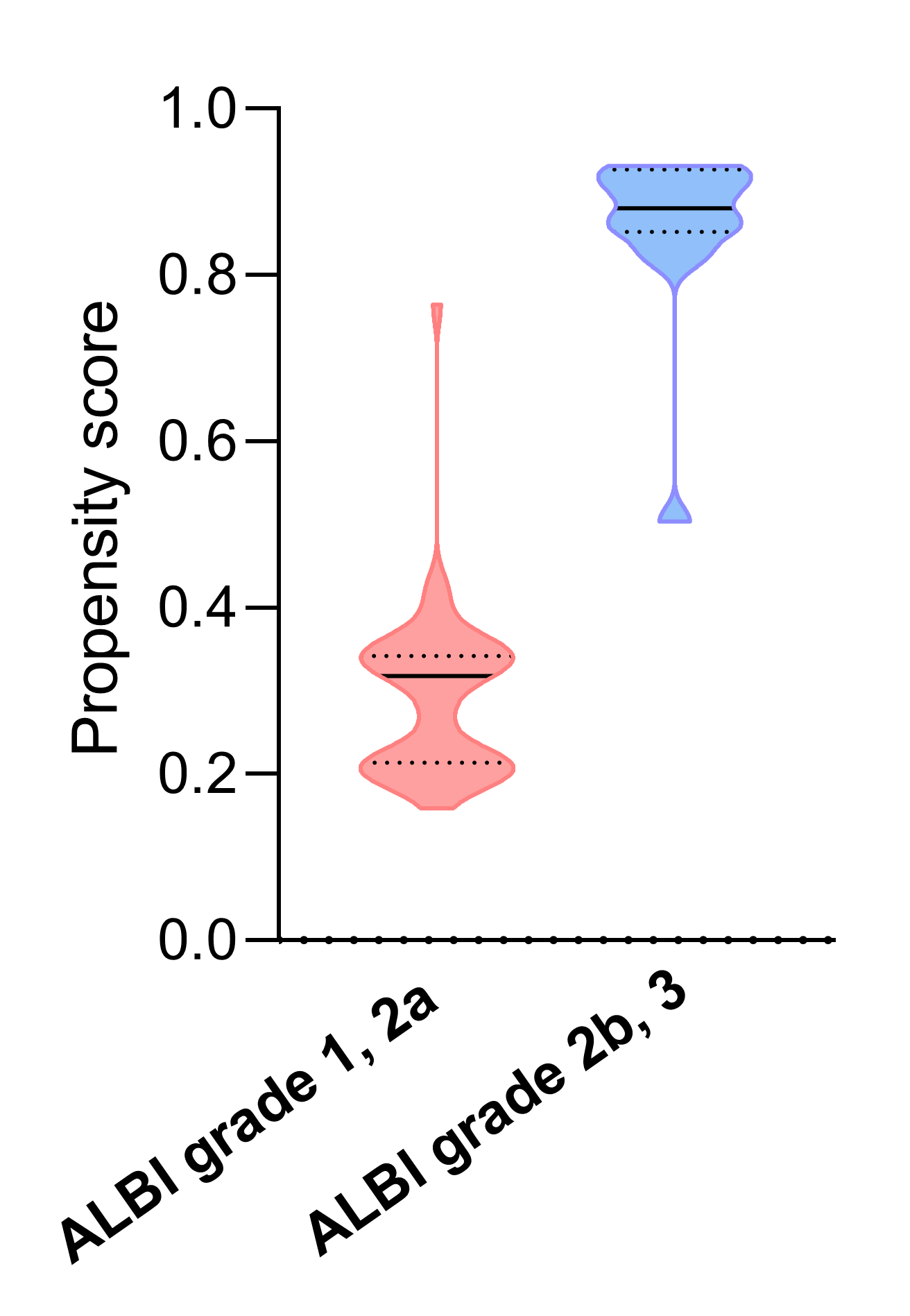


a

b

c


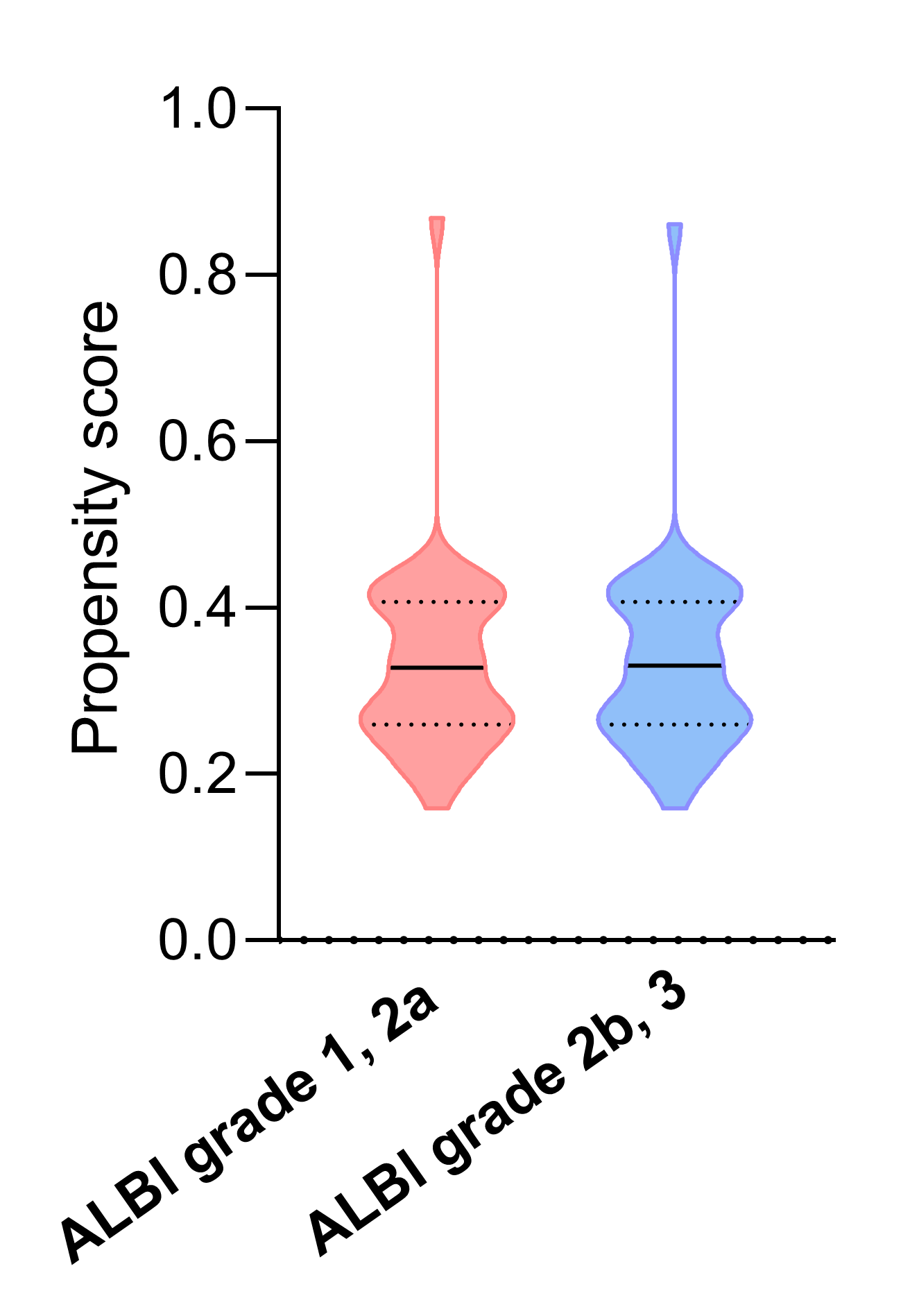


d


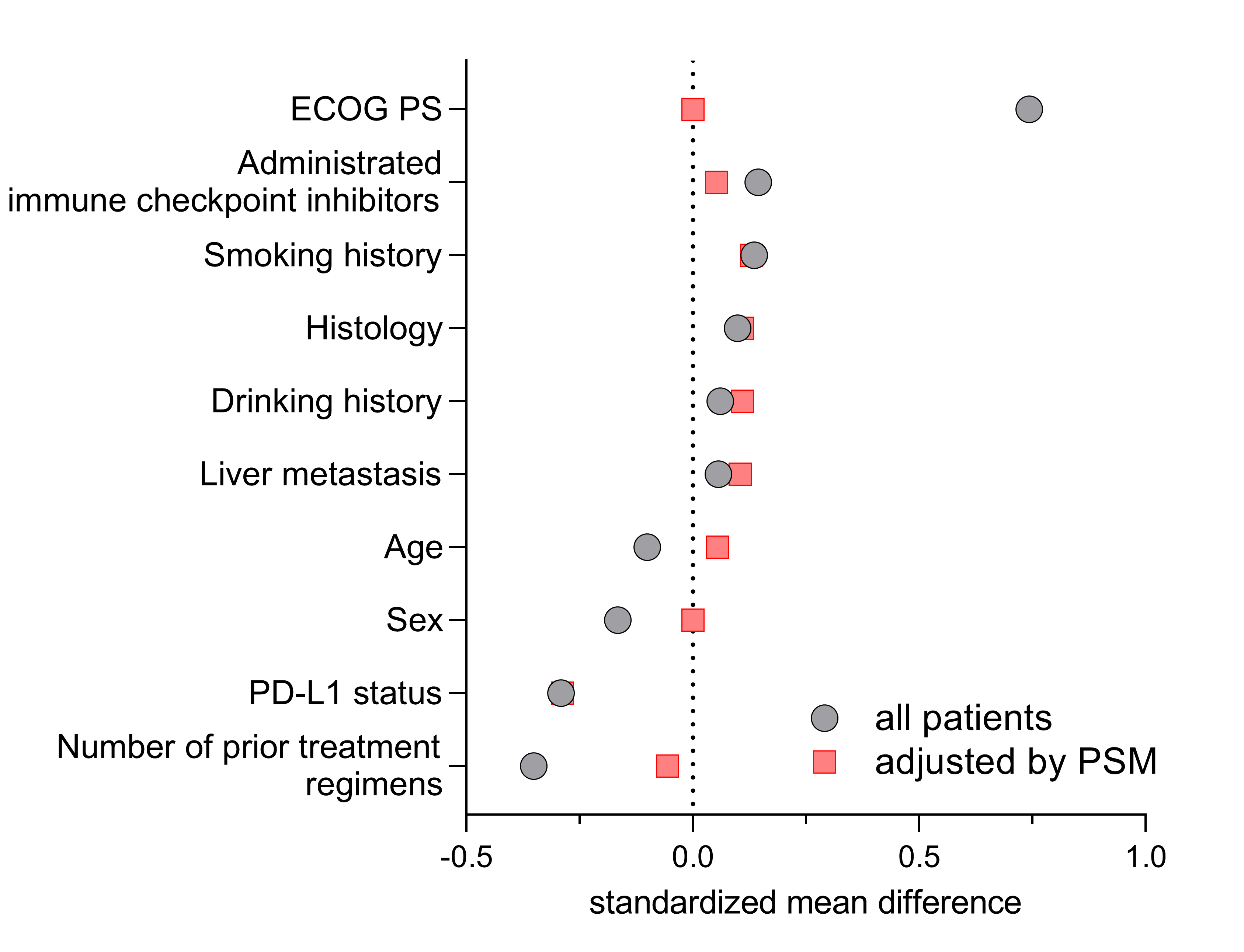


Supplementary Fig. 3

Comparison of propensity score and standardized mean differences before and after propensity score matching. The propensity score was calculated using the logistic regression model using the following baseline characteristics as covariates: age, sex, liver metastasis, drinking history, number of prior treatment regimens, and performance status. (a) Propensity score in all patients, and (b) propensity score among the patients selected by one-to-one matching between the ALBI grade 1, 2a group and ALBI 2b, 3 groups with the nearest neighbor matching. (c) The propensity score plotting of the patients excluded from the analysis. The solid line shows medians, and the dotted line shows quartiles. (d) The standardized mean difference before and after adjusting the patient background by propensity score analysis.

ECOG PS: Eastern Cooperative Oncology Group Performance Status, PD-L1: programmed cell death ligand-1, PSM: propensity score matching

**Supplementary Fig. 4**


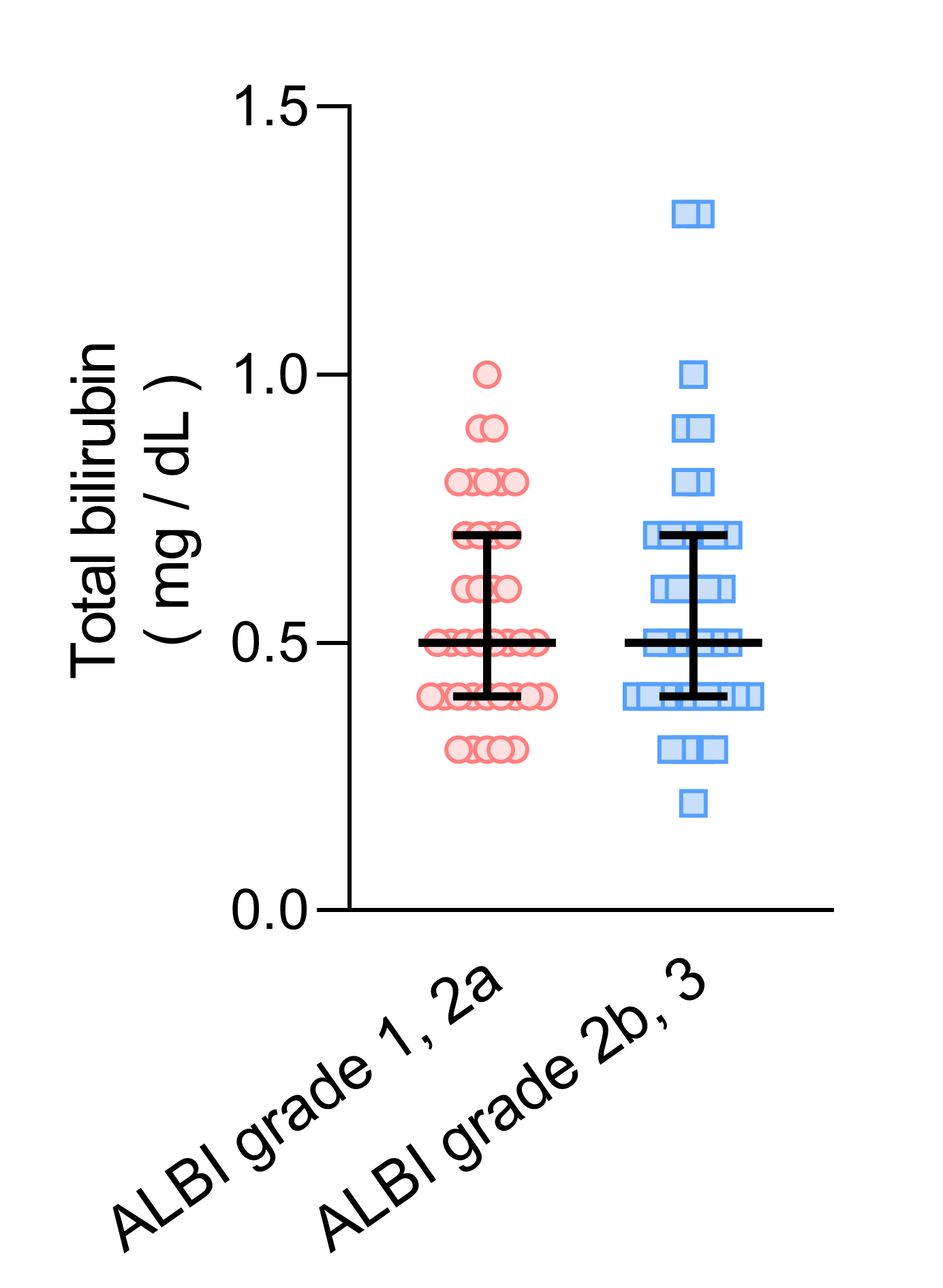

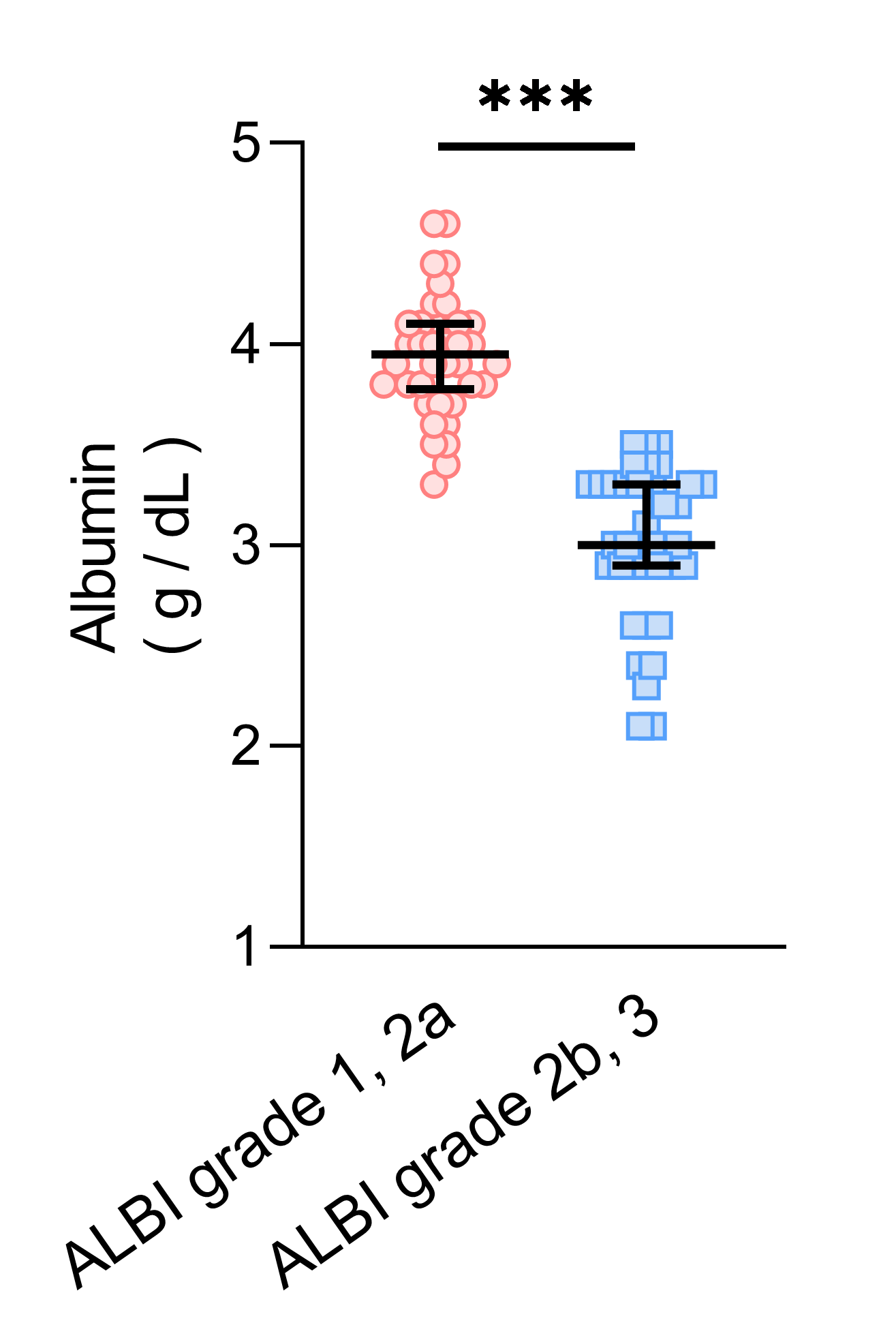

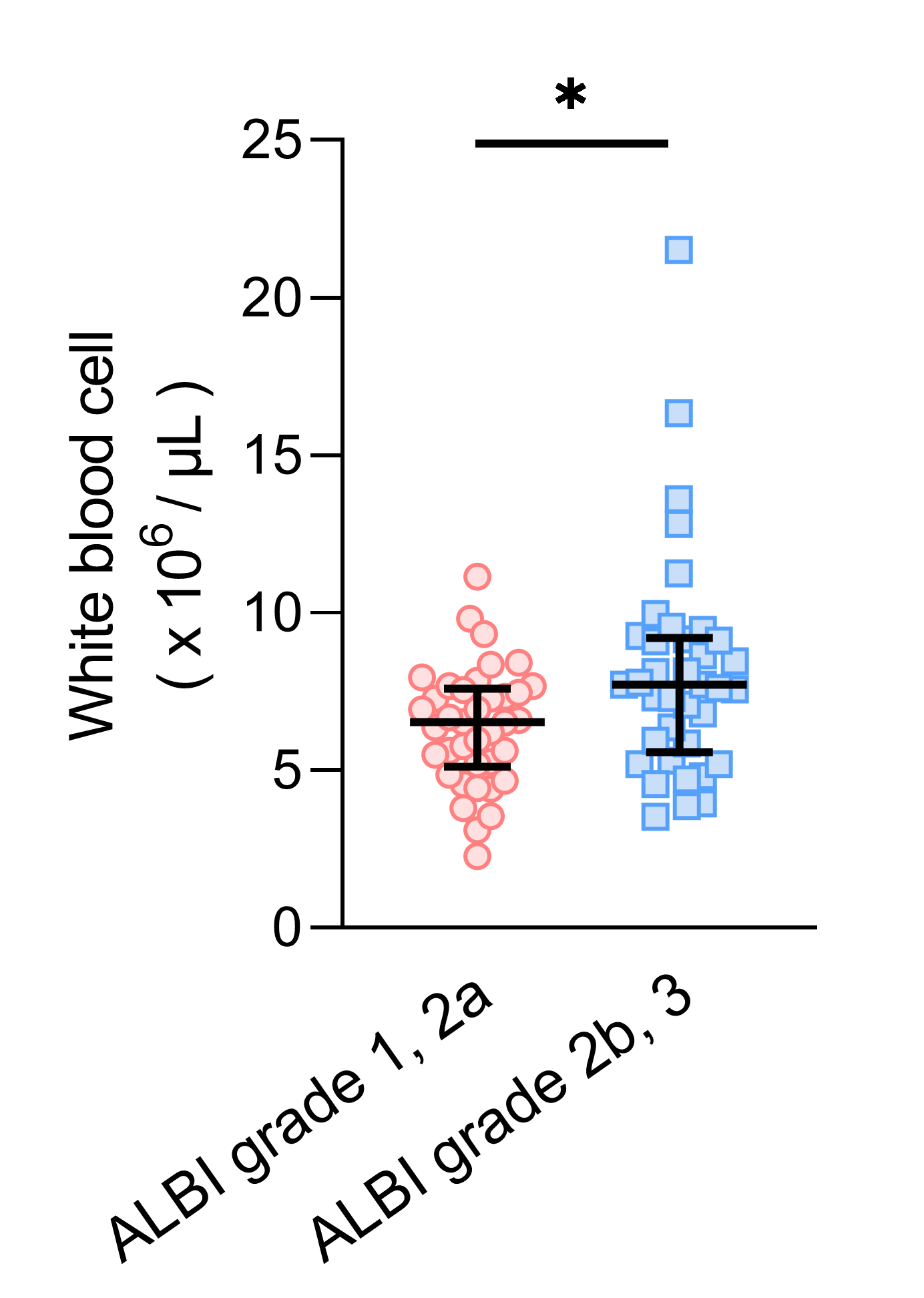

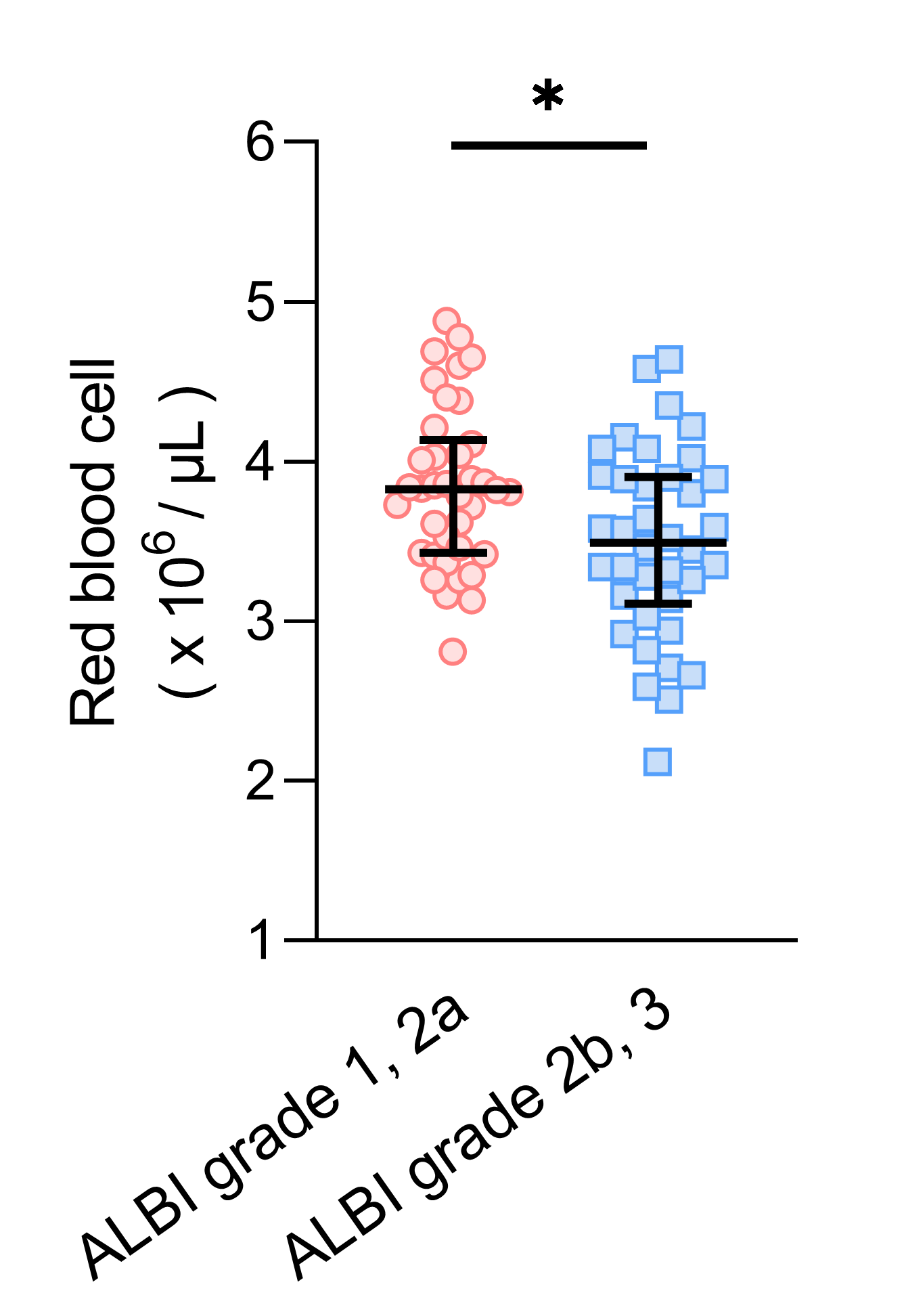

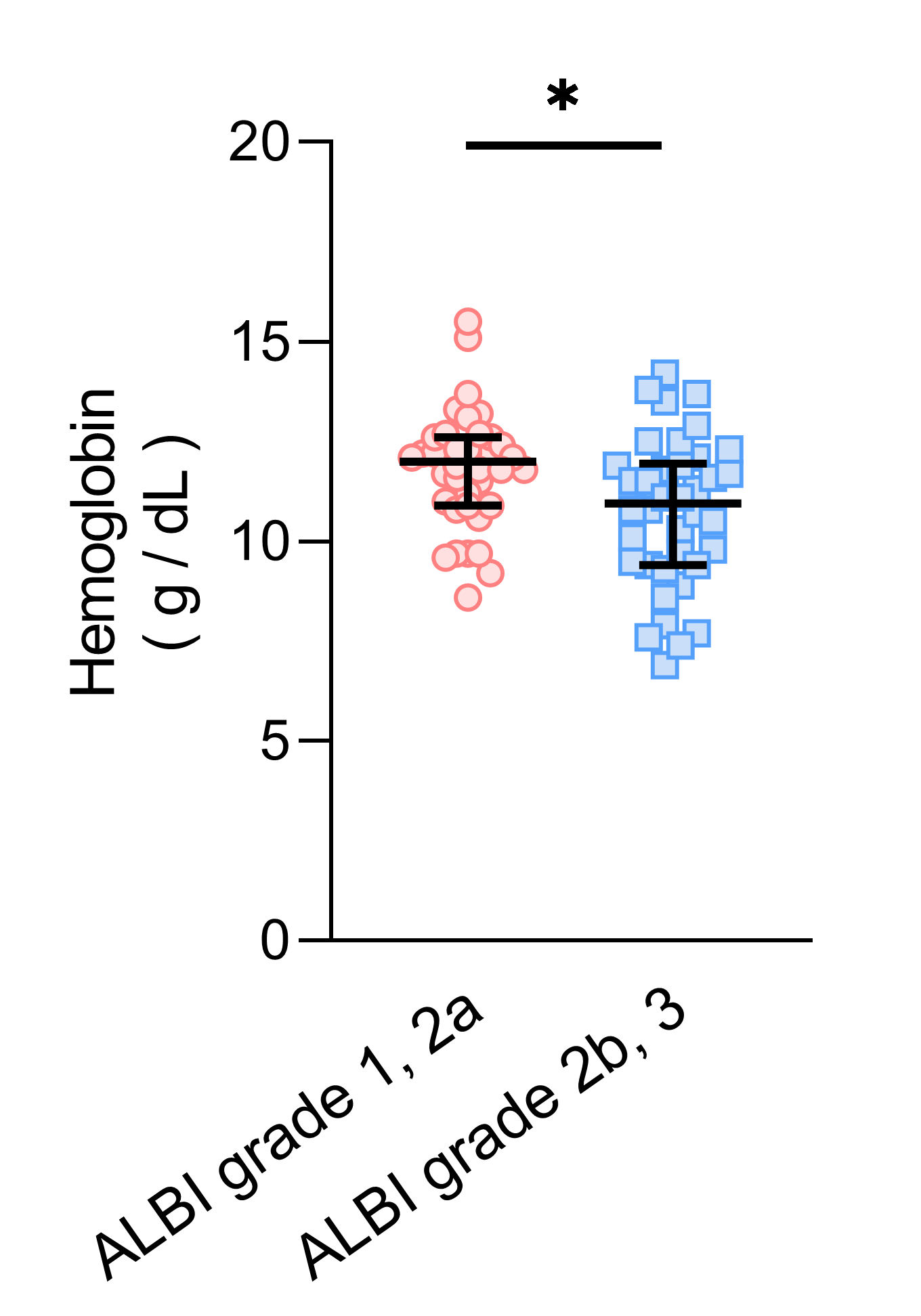

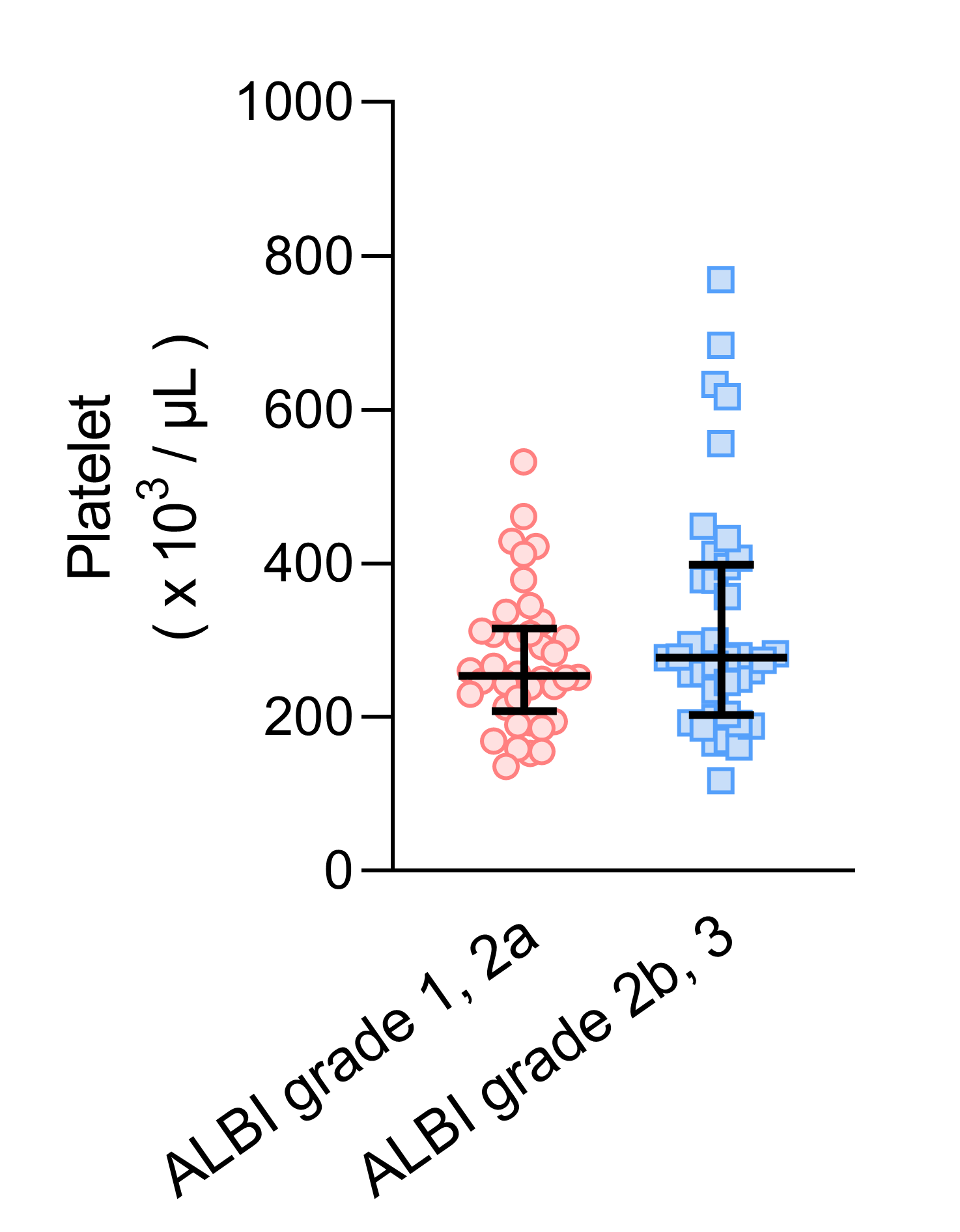

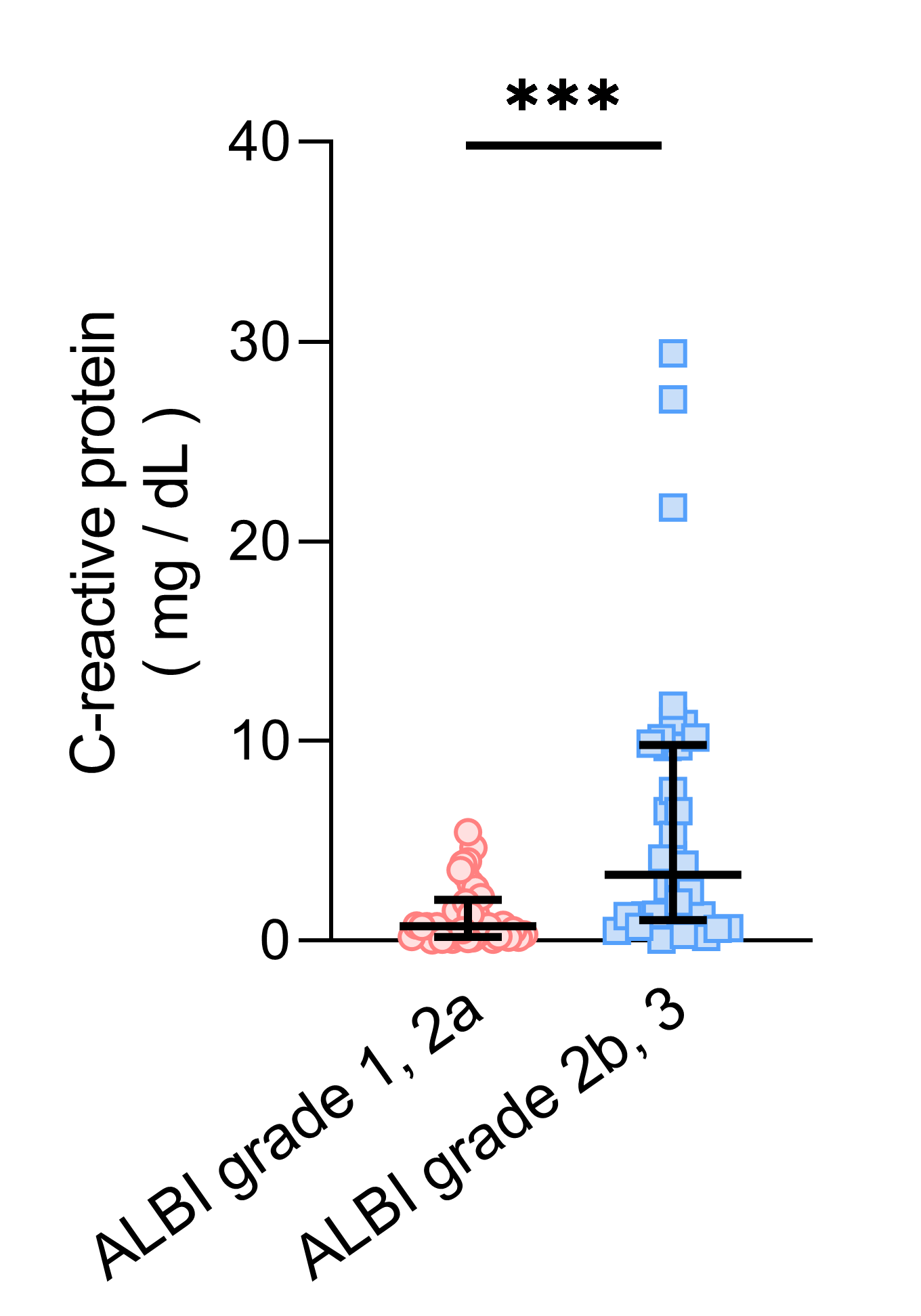

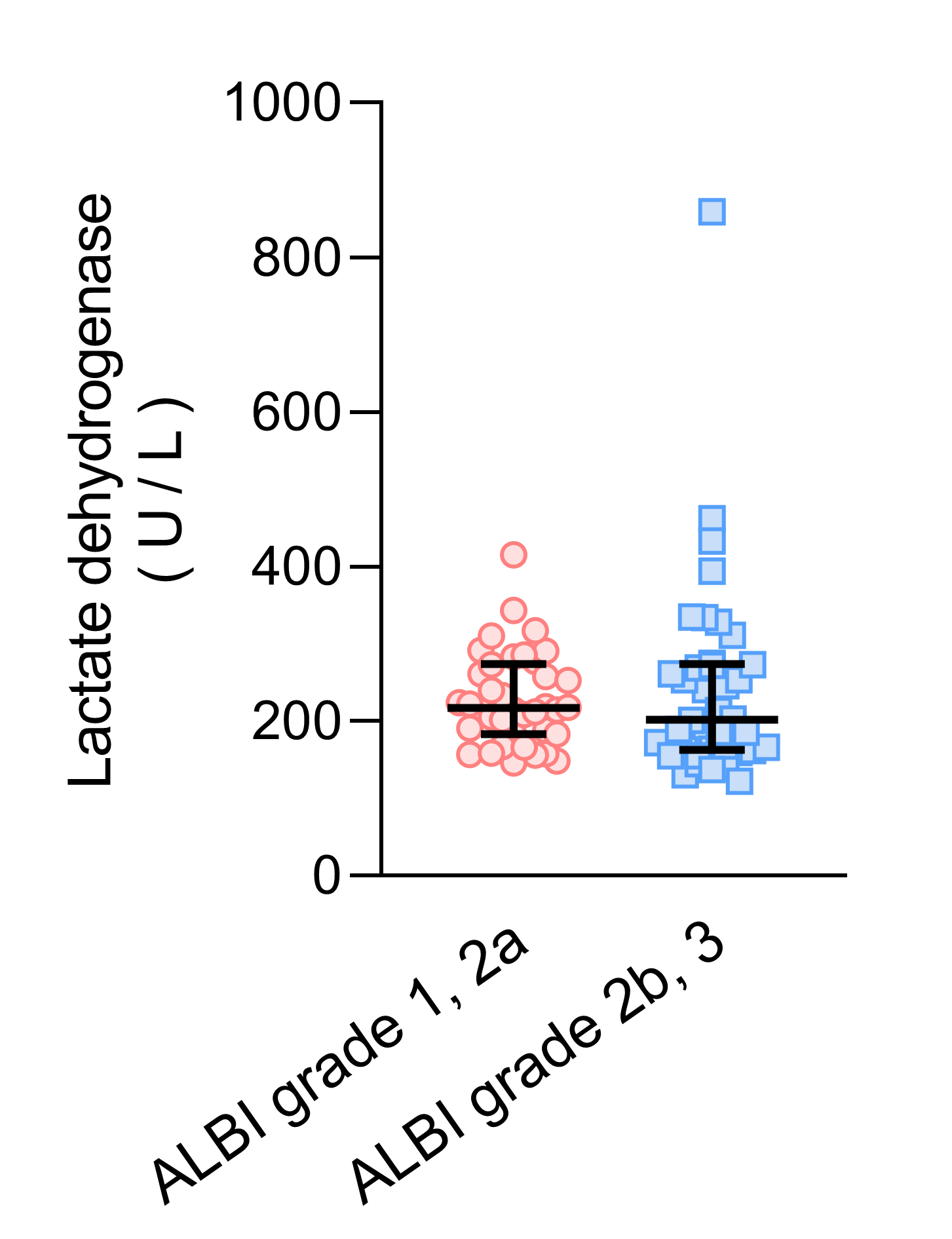

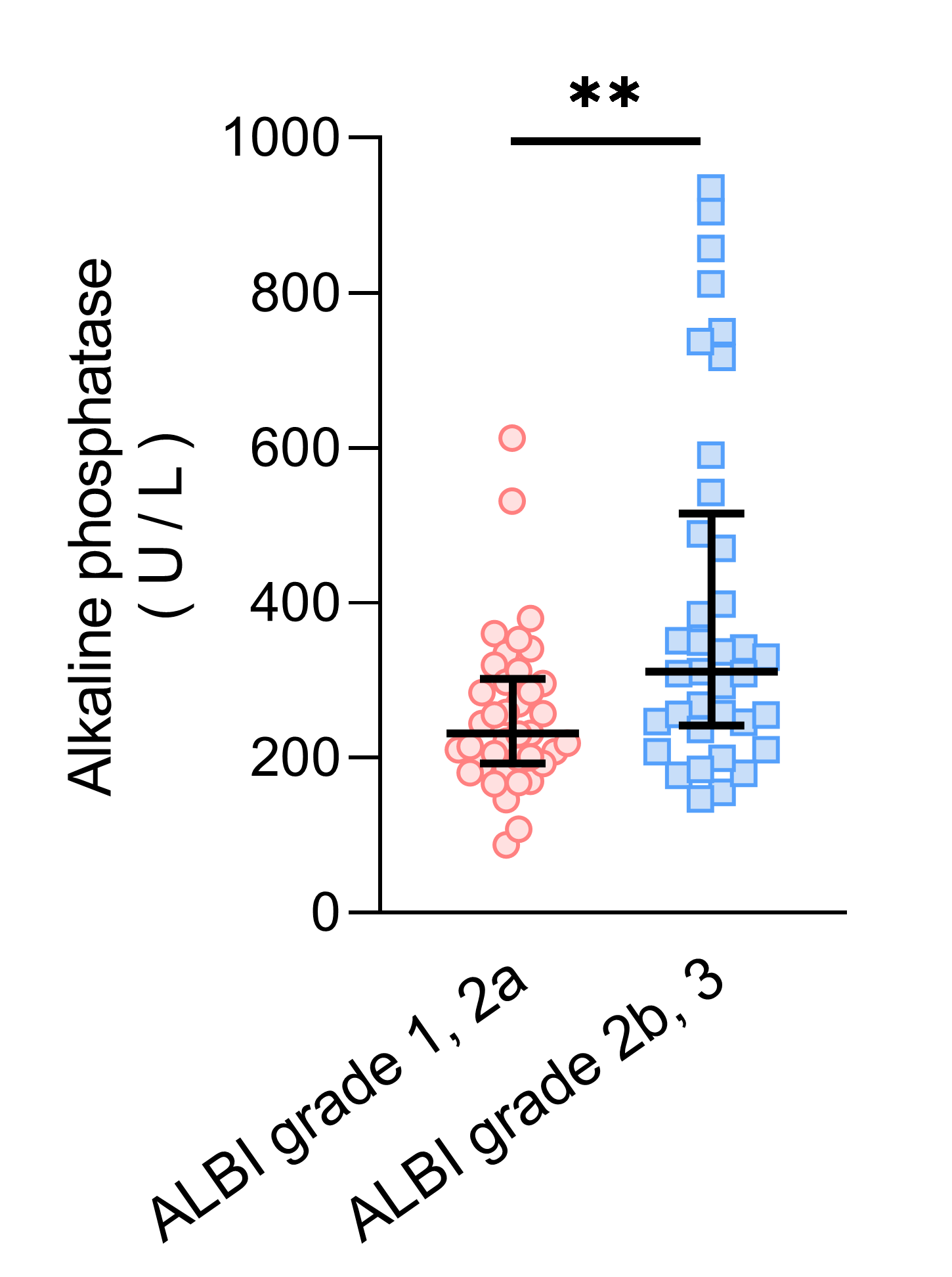

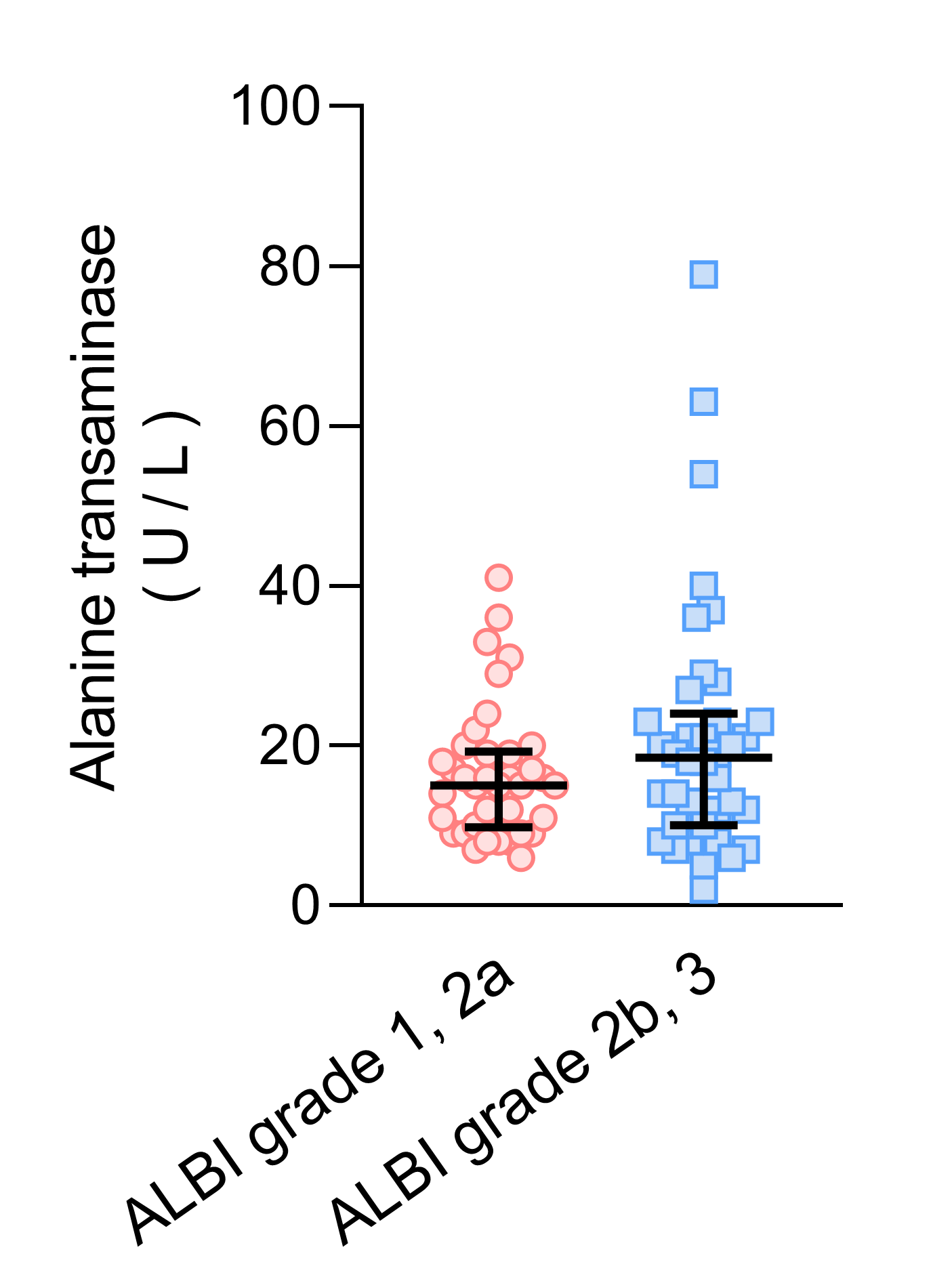

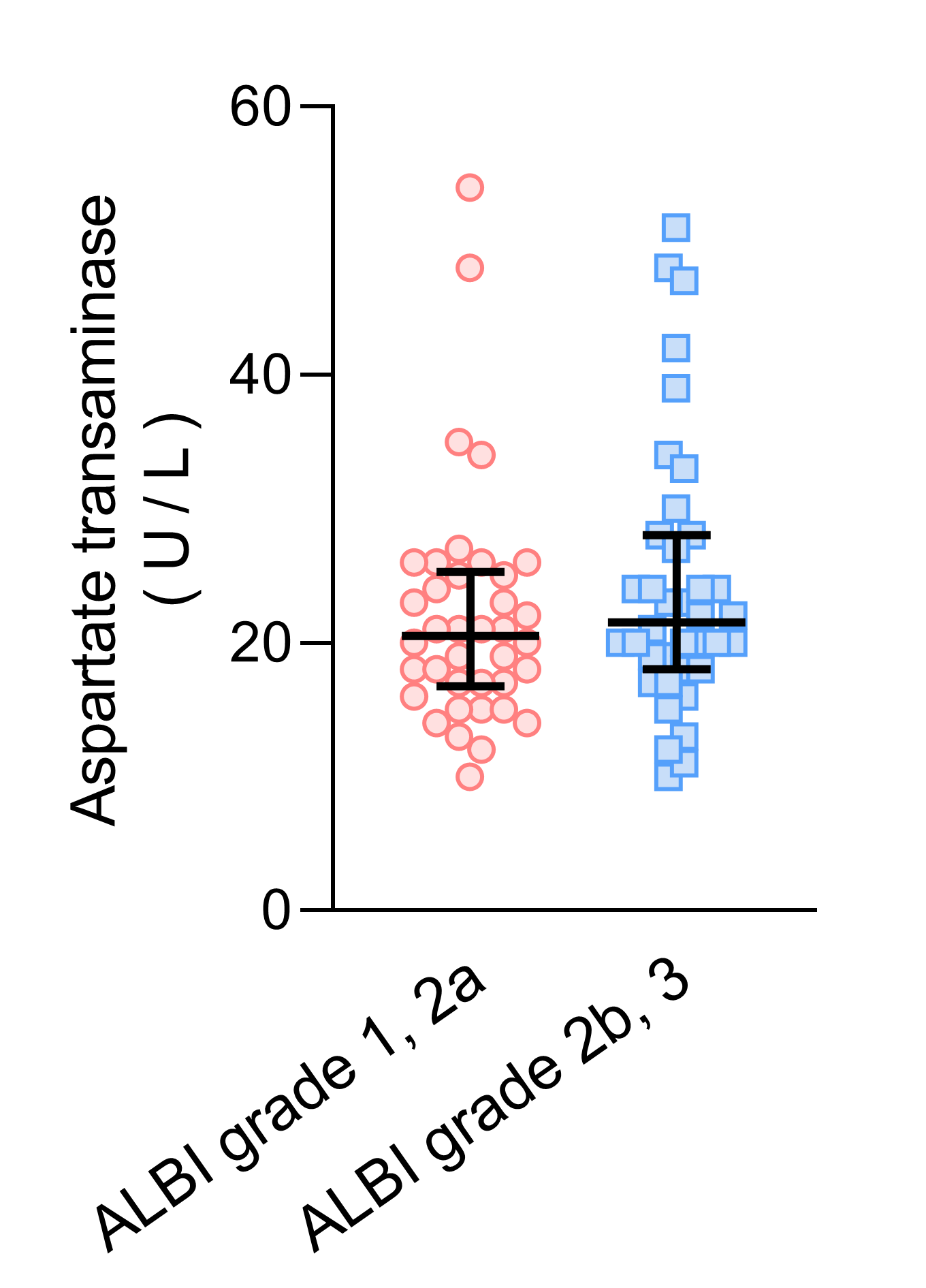


c

a

b

Supplementary Fig. 4

Pretreatment laboratory data of 38 paired patients were extracted by a propensity score matching analysis, according to albumin-bilirubin (ALBI) grade dichotomization. All data are shown as median with interquartile range. Statistical analysis was performed using the Mann-Whitney U test. * p < 0.05, ** p < 0.01, *** p < 0.005

**Supplementary Fig. 5**


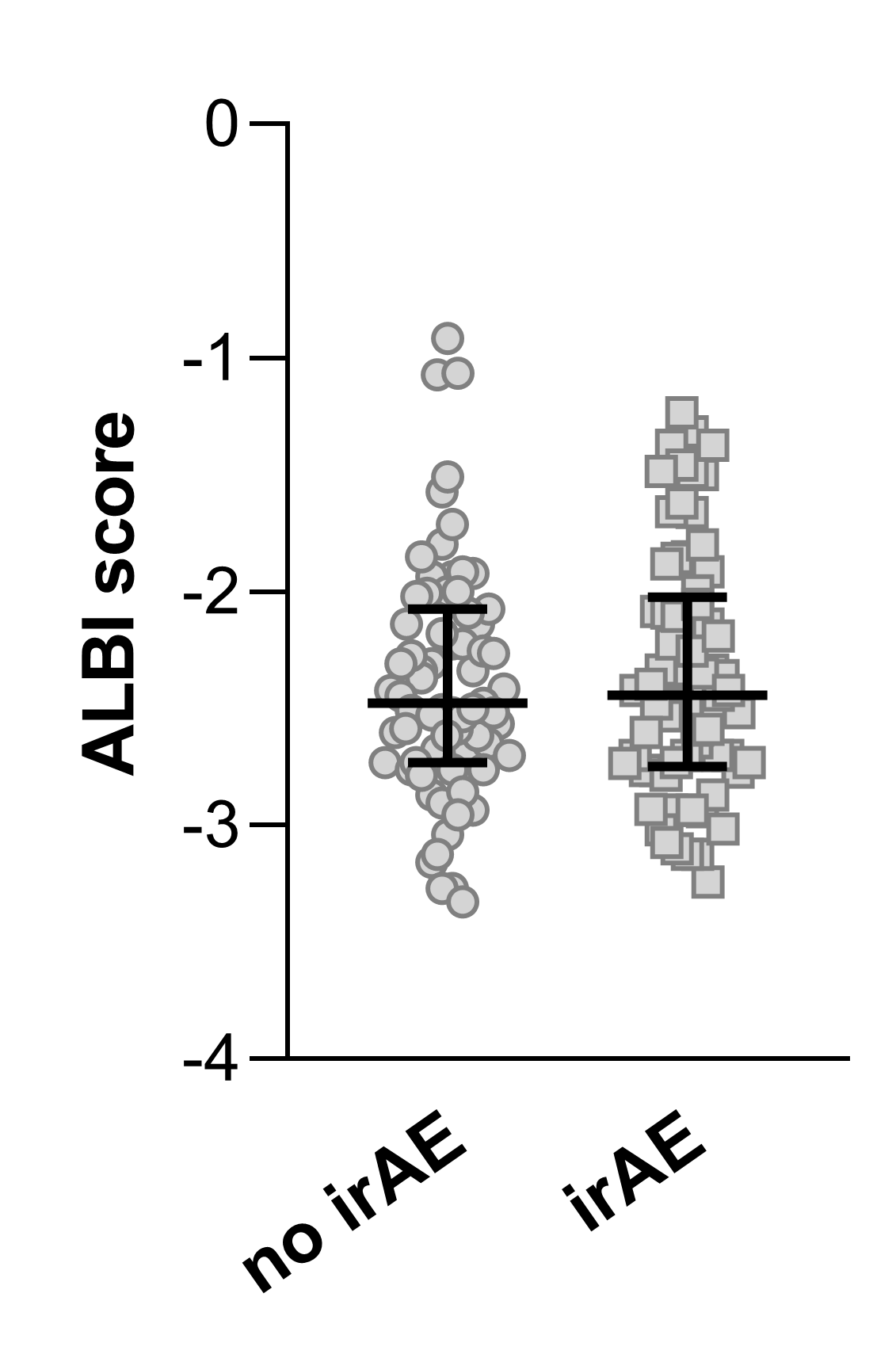

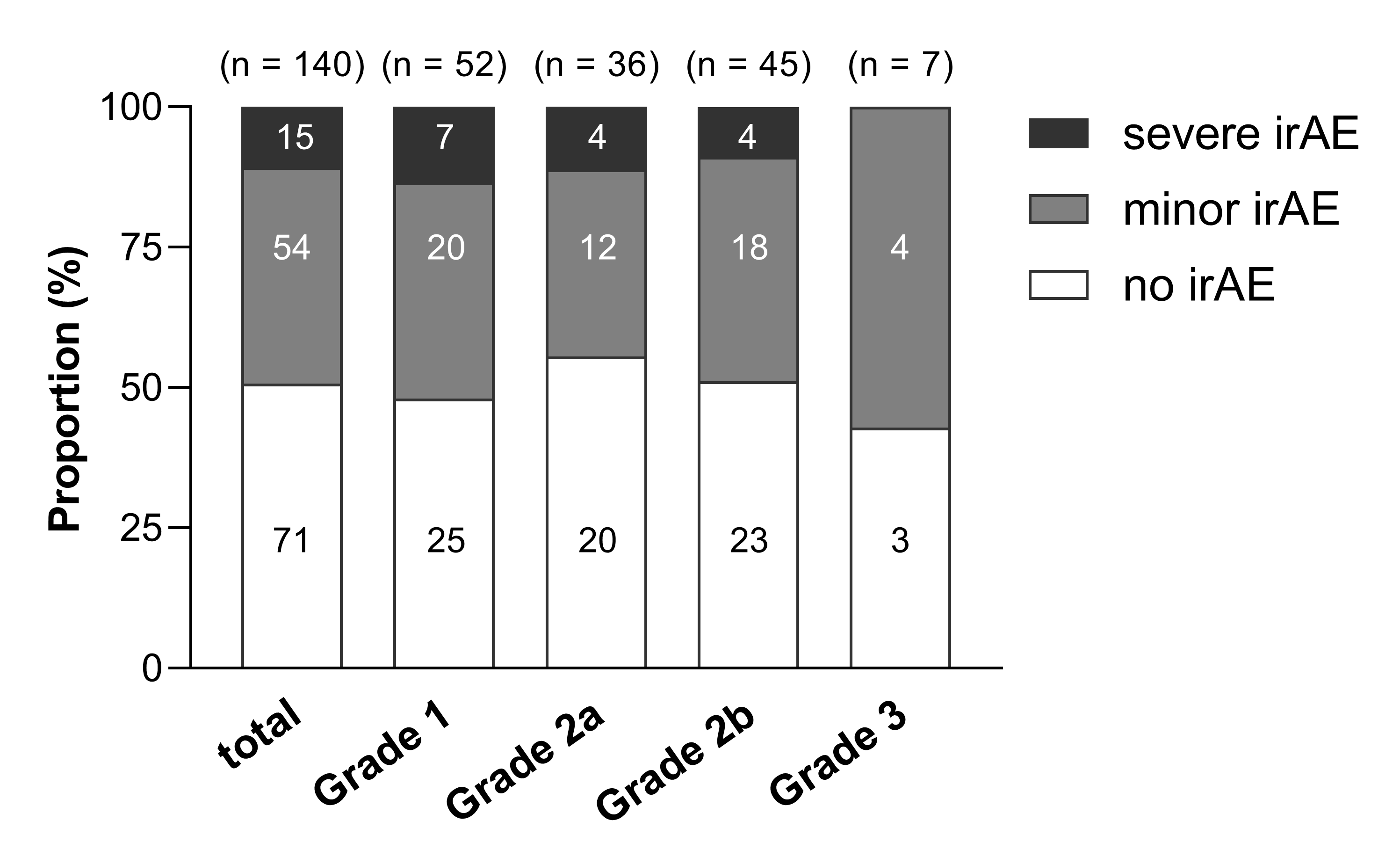


a

b

Supplementary Fig. 5

Association of the pretreatment albumin-bilirubin (ALBI) score and grade with the incidence of immune-related adverse events including its severity.
